# Supplementary material for: GATA2 Is Dispensable for Specification of Hemogenic Endothelium but Promotes Endothelial-to-Hematopoietic Transition
Source: Stem Cell Reports. 2018 May 31;11(1):197–211. doi: 10.1016/j.stemcr.2018.05.002 (PMC6066910; doi:10.1016/j.stemcr.2018.05.002)
Supplement: Document S1. Supplemental Experimental Procedures, Figures S1–S6, and Tables S1–S3 [file mmc1.pdf]

Stem Cell Reports, Volume *11*

Supplemental Information

GATA2 Is Dispensable for Specification of Hemogenic Endothelium but Promotes Endothelial-to-Hematopoietic Transition

HyunJun Kang, Walatta-Tseyon Mesquitta, Ho Sun Jung, Oleg V. Moskvin, James A. Thomson, and Igor I. Slukvin

Supplementary Information

Kang et al., GATA2 is Dispensable for Specification of Hemogenic Endothelium but Required for Endothelial-to-Hematopoietic Transition

Supplemental Experimental Procedures

Construction of inducible hGATA2-expression vector

TET response element (TRE) containing sequence of seven tetracycline operators were connected with mouse beta globin minimal promoter (mBGMP). mBGMP-TRE was inserted into PiggyBac (PB) plasmid DNA, generating a doxycycline-inducible PB-ipKTRE construct. CDS (coding domain sequence) of the human *GATA2* gene with a HA-tag in the 5'-region was connected with P2A followed by EGFP as a reporter, generating HA-GATA2-P2A-EGFP fragment, which expresses *GATA2* and *EGFP* in a bicistronic manner. Then, the HA-hGATA2-P2A-EGFP fragment was inserted into the mBGMP-TRE construct, thus generating the PB- mBGMP-TRE-HA-hGATA2-P2A-EGFP (PB-iGATA2 hereafter) construct (Figure S1). As one of the trans-activating components for TRE, an optimized form of the CDS of reverse tetracycline-controlled transactivator (*M2rtTA*) was put under a EF1alpha promoter followed by T2A and puromycin resistance (*PuroR*) gene as a selection marker, generating EF1alpha-M2rtTA-T2A-PuroR fragment, which was then inserted into PB plasmid DNA (PB-EF1alpha-M2rtTA-T2A-PuroR or PB-M2rtTA) construct (Figure S1).

Generation of intact GATA2-inducible H1 hESC (iG2$^{+/+}$) line

H1 cells growing on Matrigel in E8 media were treated with 10 μM ROCK inhibitor (ROCKi; Tocris Y-27632) 1 hour prior to nucleofection and detached by TrypLE Select (LifeTech) and singularized by pipetting. Approximately 5×10^5 cells of the single cell suspension were resuspended in 100 μL of Human Stem Cell Nucleofector Solution 1 (Lonza) containing 18 uL of Supplement 1 solution (Lonza), 5 μg of PB-iGATA2 construct, 5 μg of PB-M2rtTA construct and 1 μg of transposase-expressing plasmid DNA. Nucleofection was carried out according to manufacturer's instruction (Lonza). After transfection, cells were replated on Matrigel with E8 media containing 10 μM ROCKi and placed in 37 °C with 5% CO_2. Fresh E8 media without ROCKi was provided daily. Puromycin was added to the media at a concentration of 0.5 - 1 μg/mL around 3 - 4 days after nucleofection. Puromycin-resistant colonies were picked and expanded for further analysis and downstream experiments. Induction of transgene (GATA2 and EGFP) expression was tested by adding DOX into culture media and detecting EGFP expression by fluorescent microscopy and flow cytometry. Finally, selected colonies are referred to as intact endogenous GATA2-inducible H1 hESC (iG2$^{+/+}$) cells.

Construction of GATA2-targeting vectors

According to the general rules (Mali et al., 2013), four crRNA sequences were designed to target introns near both ends of exon 2 coding signal peptides and transactivation domain (crRNA1 & 2), and exon 5 coding zinc finger motifs for DNA

binding (crRNA3 & 4) with the goal to excise both exons (Figure 1B). These crRNAs were PCR-amplified and inserted downstream of U6 polymerase III promoter and upstream tracrRNA sequence in gRNA expression vector, MLM3636 (Addgene plasmids 43860 from Keith Joung), generating gRNA1-4 from crRNA1-4, respectively. For Cas9 protein, mammalian codon-optimized Cas9-coding plasmid DNA, JDS246 (Addgene plasmids 43861 from Keith Joung), was used.

Generation of endogenous GATA2 knockout exogenous GATA2-inducible (iG2$^{-/-}$) H1 hESC line

One hour prior to nucleofection, the iG2$^{+/+}$ cells growing on Matrigel in E8 were treated with 10 μM ROCKi, detached by TrypLE Select (LifeTech), and singularized by pipetting. 1×10^6 cells were then resuspended in 100 μL of Human Stem Cell Nucleofector Solution 1 containing 18 μL of Supplement 1 solution and 1.5 μg each of the gRNAs (gRNA1-4) and 20 μg of JDS246, and then transfected according to the manufacturer instruction (Lonza). Transfected cells were cultured on Matrigel-coated plate with E8 containing 10 μM ROCKi at 37 °C with 5% CO2. Fresh E8 media without ROCKi was provided every day. Four to five days later, single colonies were picked up and expanded. To identify and confirm a biallelic mutation in *GATA2* gene, genomic DNAs were extracted from expanded individual clones and PCR-amplified with primers specifically binding to targeting areas (Table S2). Selected mutant clones were subjected to single cell sorting using FACSAria II (BD Biosciences) to obtain single cell-derived colonies, thus ensuring the homogeneity of mutant cell lines. Genomic DNAs were extracted and PCR-amplified from single cell-derived lines to amplify and clone PCR products into a T vector for sequencing. From the sequencing results, two endogeneous GATA2 knockout exogenous GATA2-inducible cell lines, iG2$^{-/-}$SC3 and iG2$^{-/-}$SC6, were obtained and used for further experiments.

Confirming pluripotency of genetically modified hESC lines

To confirm pluripotency, flow cytometry analysis with anti-SSEA4 (Stemgent, Cambridge, MA), anti-TRA-1-60, anti-TRA-1-81 (Stemgent, MA), and anti-OCT4, anti-NANOG, and anti-SOX2 antibodies (BD Biosciences, NJ) against pluripotent markers was performed. For teratoma formation, hESCs were harvested in 100 μL of 30% Matrigel in DMEM/F12 basal media, and then injected subcutaneously into the hind leg of NOD.Cg-Prkdcscidll2rgtm1wjl/SzJ mice (The Jackson Laboratory, ME). Around 8 – 12 weeks post-injection, teratomas were collected for microscopic examination.

MACS sorting

Cells of interest that were harvested from whole differentiation cultures were subjected to MACS with the appropriated flourochrome-conjugated antibodies and corresponding magnetic microbeads. The purity of isolated fractions as verified by flow cytometry was more than 95%.

Hemangioblast and hematopoietic CFC assays

Hemangioblast assay was performed in a serum-free semisolid medium supplemented with FGF2 as previously described (Vodyanik et al., 2010). Hematopoietic CFCs were detected using H4436 MethoCult (Stem Cell Technologies) according to the manufacturer.

T cell differentiation

$CD43^+$ hematopoietic progenitors were sorted from day 8 or 9 of differentiation by MACS, and then plated on a monolayer of OP9 expressing dim/modest level of human DLL4 ($OP9^{dim}$) (Kaimakis et al., 2016) in initial T cell differentiation media (IniTDM) consisting of alpha-MEM (GIBCO) supplemented with 20% FBS (Hyclone), 50 μg/mL of ascorbic acid, 50 ng/mL FLT3L, 50 ng/mL SCF, and 20 ng/mL IL-7. The second week, T cell differentiation media was composed of alpha-MEM supplemented with 20% FBS, 50 μg/mL ascorbic acid, 20 ng/mL FLT3L, 20 ng/mL SCF, 5 ng/mL IL-7; and the third week T cell differentiation media consisted of alpha-MEM supplemented with 20% FBS, 50 μg/mL ascorbic acid, 10 ng/mL FLT3L, 20 ng/mL SCF, 5 ng/mL IL-7, 25 ng/mL IL-2. Differentiating cells were harvested using 1 mg/mL of collagenase IV (GIBCO) solution and transferred to a fresh monolayer of OP9-DLL4 weekly with the appropriate T cell differentiation media added once per week. At days 21 - 25, whole cells were harvested and analyzed by flow cytometry.

NK cell differentiation

$CD43^+$ cells from day 8 or 9 of differentiation cultures were isolated by MACS and plated on a monolayer of $OP9\text{-}DLL4^{dim}$ cells in NK cell differentiation media (NKDM) consisting of alpha-MEM supplemented with 20% Hyclone FBS, 10 ng/mL FLT3L, 40 ng/mL SCF, and 25 ng/mL IL-7. One week later, cells were transferred onto a fresh monolayer of $OP9\text{-}DLL4^{dim}$ cells in NKDM with 10 ng/mL IL-2 and 5 ng/mL IL-15. Fresh NKDM containing IL-2 and IL-15, but without IL-3, was provided every three days. Cells were harvested at days 21-25 and analyzed by flow cytometry with CD94 and CD56 after gating $CD45^+$ cells.

Flow cytometry and FACS

Flow cytometry was performed using MACSQuant® Analyzer 10 (Miltenyi Biotec) and the following antibodies: CD4-APC (RPA-T4), CD31-FITC (WM59), CD41a-APC (HIP8), CD43-APC (1G10), CD45-PE (5B1), CD73-PE (AD2), CD94-FITC (HP-3D9), CD235a-APC (GA-R2), KDR-PE (89106), and PDGFRa-PE (aR1) from BD Biosciences, CD8-Vioblue (BW135), CD43-APC-Vio770 (DF-T1), CD45-PE-Vio770 (5B1), CD144-Vioblue (REA199), and DLL4-PE-Vio770 (MHD4-46) all from Miltenyi Biotec, CD56-PerCP (HCD56) from BioLegend, and APLNR-APC (72133) from R&D Systems. Appropriate isotype-matched mouse monoclonal antibodies as flourochrome controls were included in order to establish a threshold for the positive cell population and subsequent subset gating. FACS Aria (BD) was used for cell sorting.

Western blot
Proteins were isolated using Pierce IP Lysis Buffer (Thermo Fisher Scientific) supplemented with protease inhibitor cocktail (Sigma) from day 5 of hematopoietic differentiation. Isolated proteins were boiled for 5 minutes before loading on SDS-PAGE gel. GATA2 was detected with anti-GATA2 polyclonal antibody (Grass et al., 2006) kindly provided by Dr. Emery H. Bresnick (University of Wisconsin). Anti-GAPDH antibody (Santa Cruz Biotechnology) served as loading control. Proteins on blotting membrane were detected by ECL detection reagent (GE Healthcare).

RNA isolation
RNA was isolated using RNeasy Mini Kit (Qiagen) from cell populations of interest at different time points during differentiation, and DNA digestion was performed using RNase-Free DNase Set (Qiagen) during RNA isolation according to the manufacturer's instruction protocol. Isolated RNAs were used for either quantitative RT-PCR or RNAseq. Details about qPCR, RNAseq and bioinformatics analysis can be found in Supplemental Methods.

Quantitative RT-PCR
First strand cDNAs were synthesized using MMLV reverse transcriptase (ClonTech) and then 2 μL of the first strand cDNAs were subject to RT-PCR with SYBR Advantage qPCR Premix (ClonTech). RNA levels were normalized to RPL13A or GAPDH levels, and then relative levels between samples of interest were calculated. Primer sequences are provided in Table S3.

Low level RNA-Seq data processing
Total RNA was isolated from the day 4 $iG2^{+/+}$ and $iG2^{-/-}$ HE, day 4+1 $iG2^{+/+}$ and $iG2^{-/-}$ HE cultured with and without DOX, day 6 $iG2^{+/+}$ and $iG2^{-/-}$ $VEC^{+}CD43^{-}CD73^{+}$ nonHE and day 8 $iG2^{+/+}$ and $iG2^{-/-}$ $CD43^{+}$ cells. RNA purity and integrity was evaluated by capillary electrophoresis on the Bioanalyzer 2100 (Agilent Technologies, Santa Clara, CA). Samples were then prepared for sequencing using the Ligation Mediated Sequencing (LM-Seq) protocol, according to the published guidelines (Hou et al., 2015). Final sample libraries were quantitated with the Life Technologies Qubit fluorometer and sequenced on the Illumina HiSeq 3000 (SY-401-3001). Base-calling and demultiplexing were completed with the Illumina bcl2fastq2 utility, v2.17.1.14. Following quality assessment and filtering for adapter molecules and other sequencing artifacts, the sequencing reads were aligned to transcript sequences corresponding to hg19 human genome annotation. Bowtie v 1.1.2 was used allowing two mismatches in a 25 bp seed and excluding reads with more than 200 alignments (Langmead et al., 2009). RSEM v 1.3.0 was used to estimate isoform or gene relative expression levels in units of "transcripts per million" (tpm), as well as posterior mean estimate of the "expected counts" (the non-normalized absolute number of reads assigned by RSEM to each isoform/gene) (Li and Dewey, 2011; Li et al., 2010). R statistical environment (R core team, 2014) was used at all stages of downstream data analysis.

Downstream RNAseq bioinformatics analysis

Testing for differential expression

The entire set of libraries was pre-normalized as a pool using median normalization routine from EBSeq package (Leng et al., 2013). For each gene, maximal counts across all samples were plotted and the genes representing the lower mode of the distribution were filtered out (only genes that have at least 40 – counts in at least 1 sample were retained, restricting the set of genomic features to 12,635. Additional median scaling was applied to the pre-filtered set of genes. Differential expression was called using EBSeq with 10 iterations. The EBSeq's default procedure of filtering low-expressed genes was suppressed by setting the QtrmCut parameter to zero. Genes with assigned value of Posterior Probability of Differential Expression above 0.95 were preliminary selected. Genes that additionally passed fold change cutoff of 1.5 were selected for network analysis.

Construction of GATA2 network

Using the known transcription-target relationships obtained by combining largely complementary data from HTRIdb (Bovolenta et al., 2012) and CellNet (Cahan et al., 2014), we generated combined sets of targets for 950 transcriptional regulators that involve 130,855 individual transcription factor (TF)-target interactions, for regulon analysis. To visualize the cascades of transcriptional regulation that involve influence of active TFs on TF-encoding genes, we restricted the overall regulatory network to TF-target relationships that involve TF-encoding target genes. The resulting "transcriptional backbone" network has 837 regulators and reduced by over an order of magnitude (12,372) individual TF-target relationships. To visualize the core of the relevant regulatory network, we first selected the regulons that a) responded with FDR below 0.01 in any direction in at least 2 out of 5 comparisons described in the text and b) are listed as both regulators and targets in the "transcriptional backbone" network. The resulting network contains 110 nodes and 983 edges. Each node represents both TF and the gene encoding that TF. The double nature of nodes is used to visualize two types of responses: change in regulon activity (estimated with the complete network of 130,855 TF-target relationships) as node color and change in mRNA of the TF-encoding gene as node size.

Gene-sharing network

To generate gene-sharing networks, we collected respective category-gene relationships (e.g. Gene Ontology Cellular Component) and restricted them to genes that were called differentially expressed in at least one of 5 comparisons (see the Results). Then, for each pair of the categories, the number of shared genes was recorded and used to map to the edge width of the resulting network.

Supplemental References

Bovolenta, L.A., Acencio, M.L., and Lemke, N. (2012). HTRIdb: an open-access database for experimentally verified human transcriptional regulation interactions. BMC Genomics *13*, 405.

Cahan, P., Li, H., Morris, S.A., Lummertz da Rocha, E., Daley, G.Q., and Collins, J.J. (2014). CellNet: network biology applied to stem cell engineering. Cell *158*, 903-915.
Grass, J.A., Jing, H., Kim, S.I., Martowicz, M.L., Pal, S., Blobel, G.A., and Bresnick, E.H. (2006). Distinct functions of dispersed GATA factor complexes at an endogenous gene locus. Mol Cell Biol *26*, 7056-7067.
Hou, Z., Jiang, P., Swanson, S.A., Elwell, A.L., Nguyen, B.K., Bolin, J.M., Stewart, R., and Thomson, J.A. (2015). A cost-effective RNA sequencing protocol for large-scale gene expression studies. Sci Rep *5*, 9570.
Kaimakis, P., de Pater, E., Eich, C., Solaimani Kartalaei, P., Kauts, M.L., Vink, C.S., van der Linden, R., Jaegle, M., Yokomizo, T., Meijer, D.*, et al.* (2016). Functional and molecular characterization of mouse Gata2-independent hematopoietic progenitors. Blood *127*, 1426-1437.
Langmead, B., Trapnell, C., Pop, M., and Salzberg, S.L. (2009). Ultrafast and memory-efficient alignment of short DNA sequences to the human genome. Genome Biol *10*, R25.
Leng, N., Dawson, J.A., Thomson, J.A., Ruotti, V., Rissman, A.I., Smits, B.M., Haag, J.D., Gould, M.N., Stewart, R.M., and Kendziorski, C. (2013). EBSeq: an empirical Bayes hierarchical model for inference in RNA-seq experiments. Bioinformatics *29*, 1035-1043.
Li, B., and Dewey, C.N. (2011). RSEM: accurate transcript quantification from RNA-Seq data with or without a reference genome. BMC Bioinformatics *12*, 323.
Li, B., Ruotti, V., Stewart, R.M., Thomson, J.A., and Dewey, C.N. (2010). RNA-Seq gene expression estimation with read mapping uncertainty. Bioinformatics *26*, 493-500.
Mali, P., Yang, L., Esvelt, K.M., Aach, J., Guell, M., DiCarlo, J.E., Norville, J.E., and Church, G.M. (2013). RNA-guided human genome engineering via Cas9. Science *339*, 823-826.
Vodyanik, M.A., Yu, J., Zhang, X., Tian, S., Stewart, R., Thomson, J.A., and Slukvin, II (2010). A mesoderm-derived precursor for mesenchymal stem and endothelial cells. Cell Stem Cell *7*, 718-729.

A

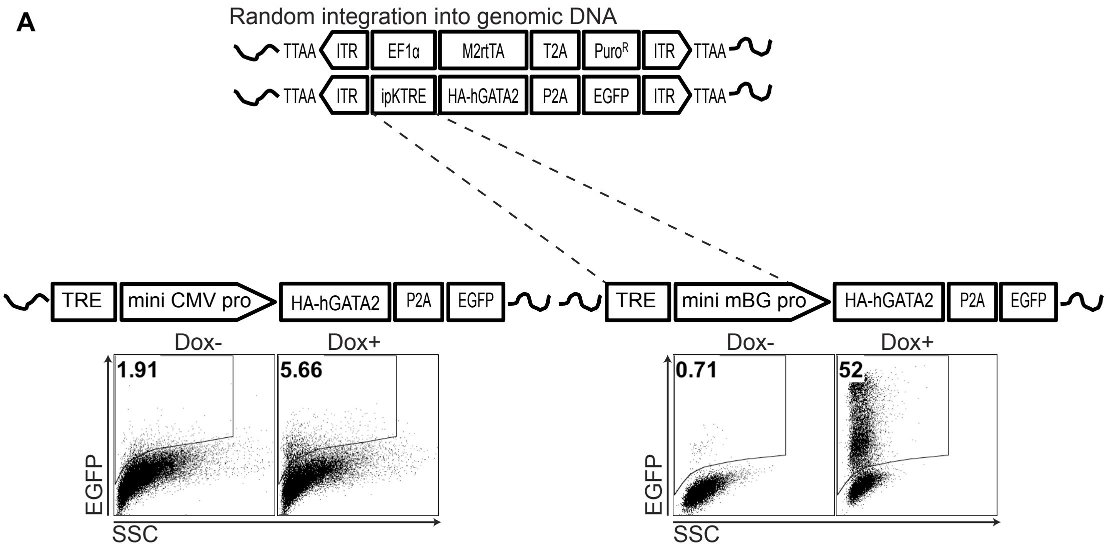

B

Product size 483 bp

```
ATTCCATGGTTCTGAGATGCTTTGATTCCCATGTGAGATTTAGCCCTCCTTGACTGAGCt
||||||||||||||||||||||||||||||||||||||||||||||||||||||||||||
ATTCCATGGTTCTGAGATGCTTTGATTCCCATGTGAGATTTAGCCCTCCTTGACTGAGCT
ggtggggactgggggtggagcgagggtcagggagggggtcgaggtgggcgtgggagTCC
||||||||||||||||||||||||||||||||||||||||||||||||||||||||||||
GGTGGGGACTGGGGGTGGAGCGAGGGTCAGGGAGGGGGGTCGAGGTGGGCGTGGGAGTCC
AGCCTGCTGACGCTGCCTTGCCCTCCCAGTCGGCCGCCAGAAGAGCCGGCACCTGTTGTG
||||||||||||||||||||||||||||||||||||||||||||||||||||||||||||
AGCCTGCTGACGCTGCCTTGCCCTCCCAGTCGGCCGCCAGAAGAGCCGGCACCTGTTGTG
CAAATTGTCAGACGACAACCACCACCTTATGGCGCCGAAACGCCAACGGGGACCCTGTCT
||||||||||||||||||||||||||||||||||||||||||||||||||||||||||||
CAAATTGTCAGACGACAACCACCACCTTATGGCGCCGAAACGCCAACGGGGACCCTGTCT
GCAACGCCTGTGGCCTCTACTACAAGCTGCACAATGTGAGTGCGCCCCGCCCCGGCCACC
||||||||||||||||||||||||||||||||||||||||||||||||||||||||||||
GCAACGCCTGTGGCCTCTACTACAAGCTGCACAATGTGAGTGCGCCCCGCCCCGGCCACC
CCGCCCCTCCCAGGGGACCTCTGCGCTTTGTGCTGCCAGGCAAGAGGCCCCAGCCACAAT
||||||||||||||||||||||||||||||||||||||||||||||||||||||||||||
CCGCCCCTCCCAGGGGACCTCTGCGCTTTGTGCTGCCAGGCAAGAGGCCCCAGCCACAAT
ATCCAGCTTGGCTTGGCTTGGGAAGCTGCTGCCCTGAGTGAGCGCCAGAAGGGCTTCCCG
||||||||||||||||||||||||||||||||||||||||||||||||||||||||||||
ATCCAGCTTGGCTTGGCTTGGGAAGCTGCTGCCCTGAGTGAGCGCCAGAAGGGCTTCCCG
TAAGAGGGGTGCCTTGCCTCTGCTCAGGAGGTGGAGCTGGCTAGGACAGGGTCTCGGACT
||||||||||||||||||||||||||||||||||||||||||||||||||||||||||||
TAAGAGGGGTGCCTTGCCTCTGCTCAGGAGGTGGAGCTGGCTAGGACAGGGTCTCGGACT
AGGGAA Genomic DNA for intron-exon5-intron
||||||
AGGGAA PCR products
```

Figure S1. Characterization of GATA2-inducible and GATA2 knockout cell lines, related to Figure 1. (A) Modification of tetracycline response element (ipKTRE), to enhance resistance to transgene silencing during differentiation. Flow cytometry dot plots after 4 days of hematopoietic differentiation show the lack of EGFP expression following DOX treatment of hESCs modified using TREminiCMV promoter. (B) Sequencing of intron-exon5-intron *GATA2* region targeted with gRNA3 and gRNA4 shows preserved structure of this region in generated iG2$^{-/-}$ cells.

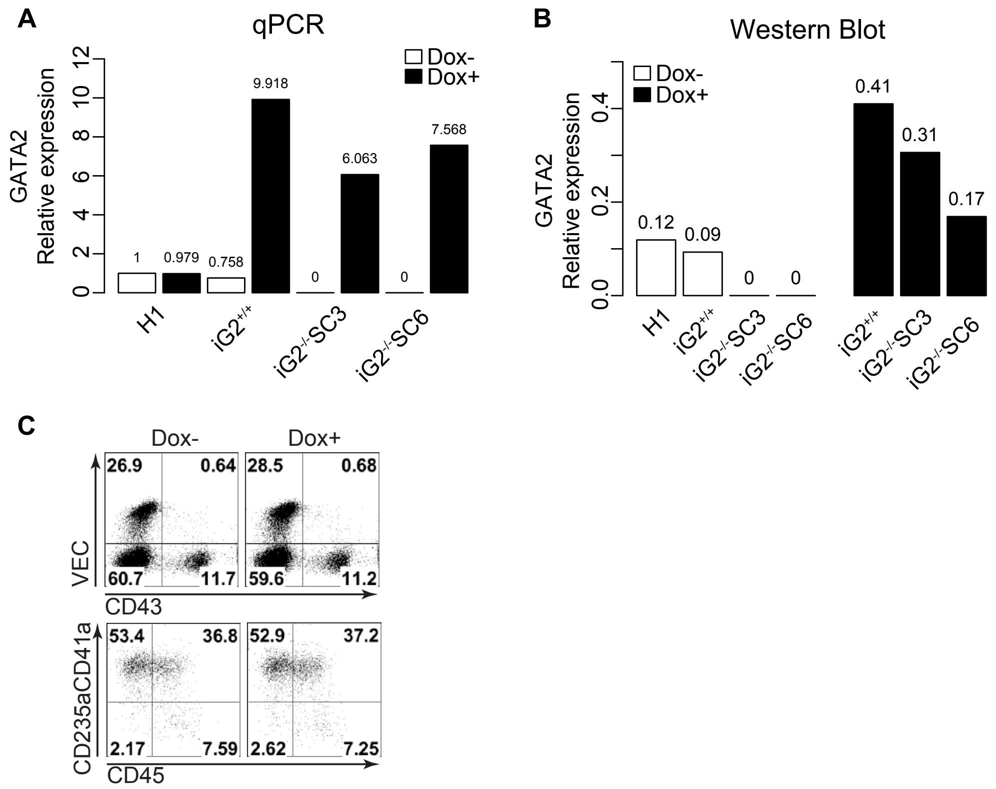

Figure S2. Analysis of DOX effect on GATA2 expression and hematopoietic differentiation, related to Figure 1. (A) qPCR analysis of GATA2 expression on day 5 of hematopoietic differentiation in cultures with or without DOX. (B) Western blot quantification of GATA2 expression on day 5 of hematopoietic differentiation in cultures treated and non-treated with DOX. H1 is wild type H1 hESCs. (C) Flow cytometric analysis blood formation on day 8 of differentiation of wild type H1 hESCs with or without DOX.

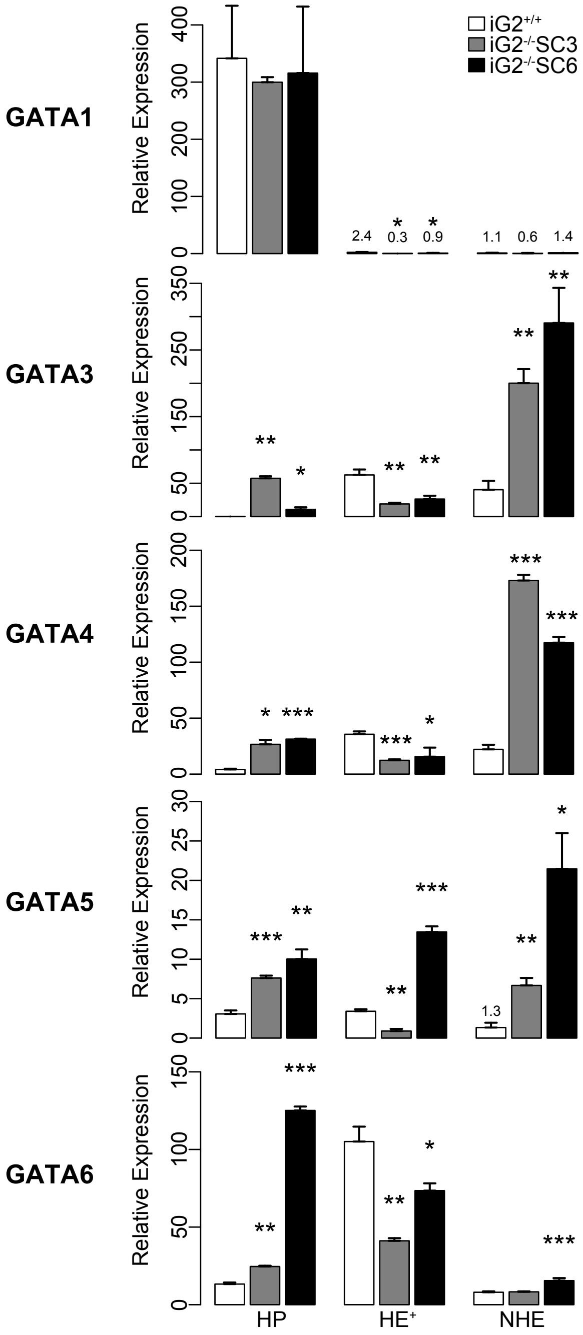

Figure S3. qPCR analysis of GATA factors in CD43+ hematopoietic progenitors on day 8 differentiation (HP), HE and non-HE, related to Figure 3. Bars show mean±SE for 3 independent experiments.

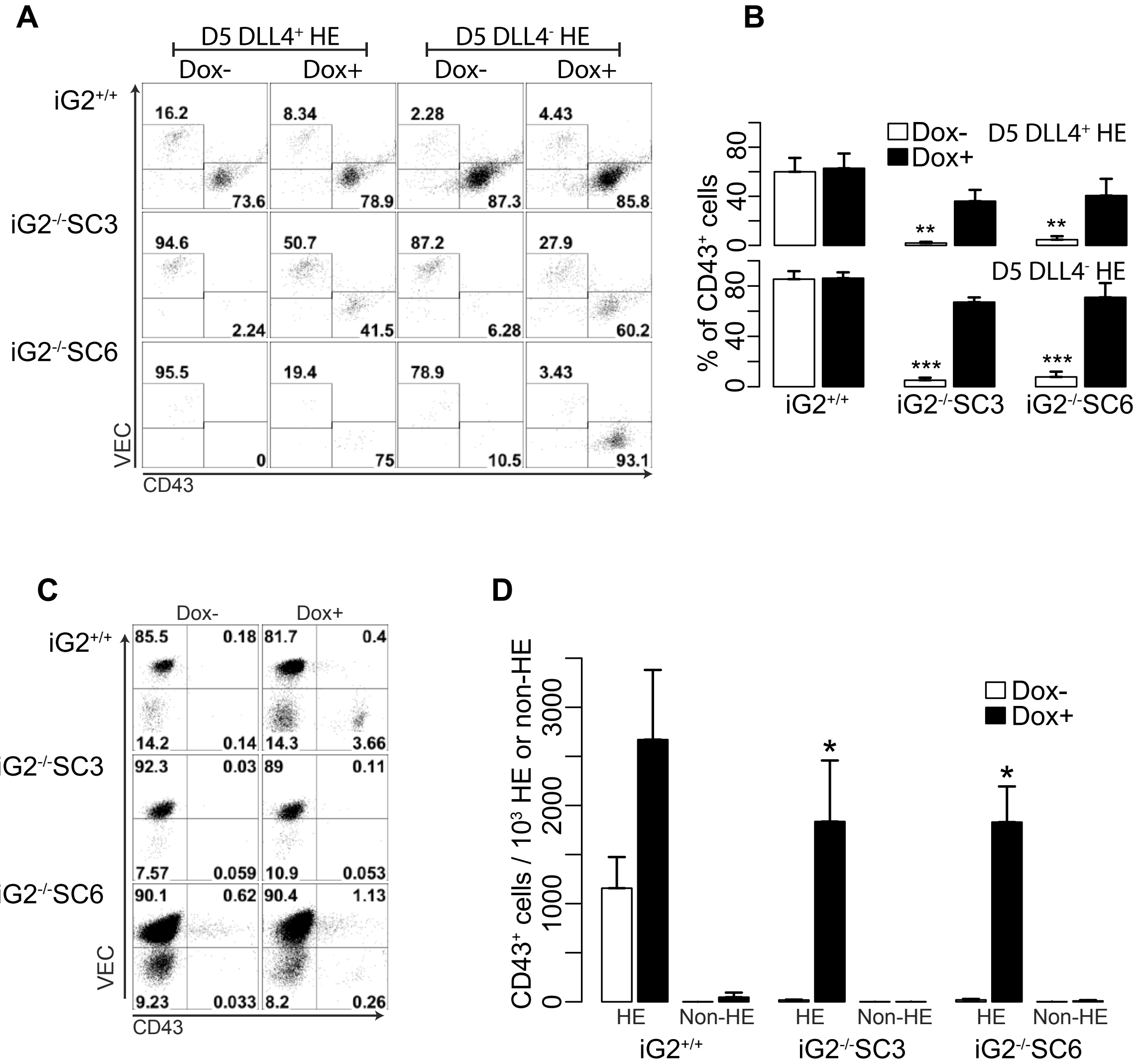

Figure S4. GATA2 effect on blood production from $DLL4^+$ and $DLL4^-$ HE and non-HE, related to Figure 5. (A) and (B) Blood forming potential of $DLL4^+$ and $DLL4^-$ HE. $DLL4^+$ and $DLL4^-$ $VEC^+CD43^-CD73^-$ HE cells were isolated by FACS on day 5 of differentiation and cultured on DLL4-OP9 stromal cells with and without DOX for 6 days. (C) Flow cytometric analysis shows the effect of DOX on blood production form $VEC^+CD43^-CD73^+$ non-HE. Non-HE were isolated from $iG2^{+/+}$ and $iG2^{-/-}$ cells and cultured in HE conditions with or without DOX added during first 2 days of secondary culture. Flow cytometric analysis was performed after 6 days of differentiation. (D) Absolute numbers of $CD43^+$ cells generated from 1000 HE and non-HE cells in DOX+ and DOX- cultures. Bars in (B) and (D) show mean±SE for 3 independent experiments.

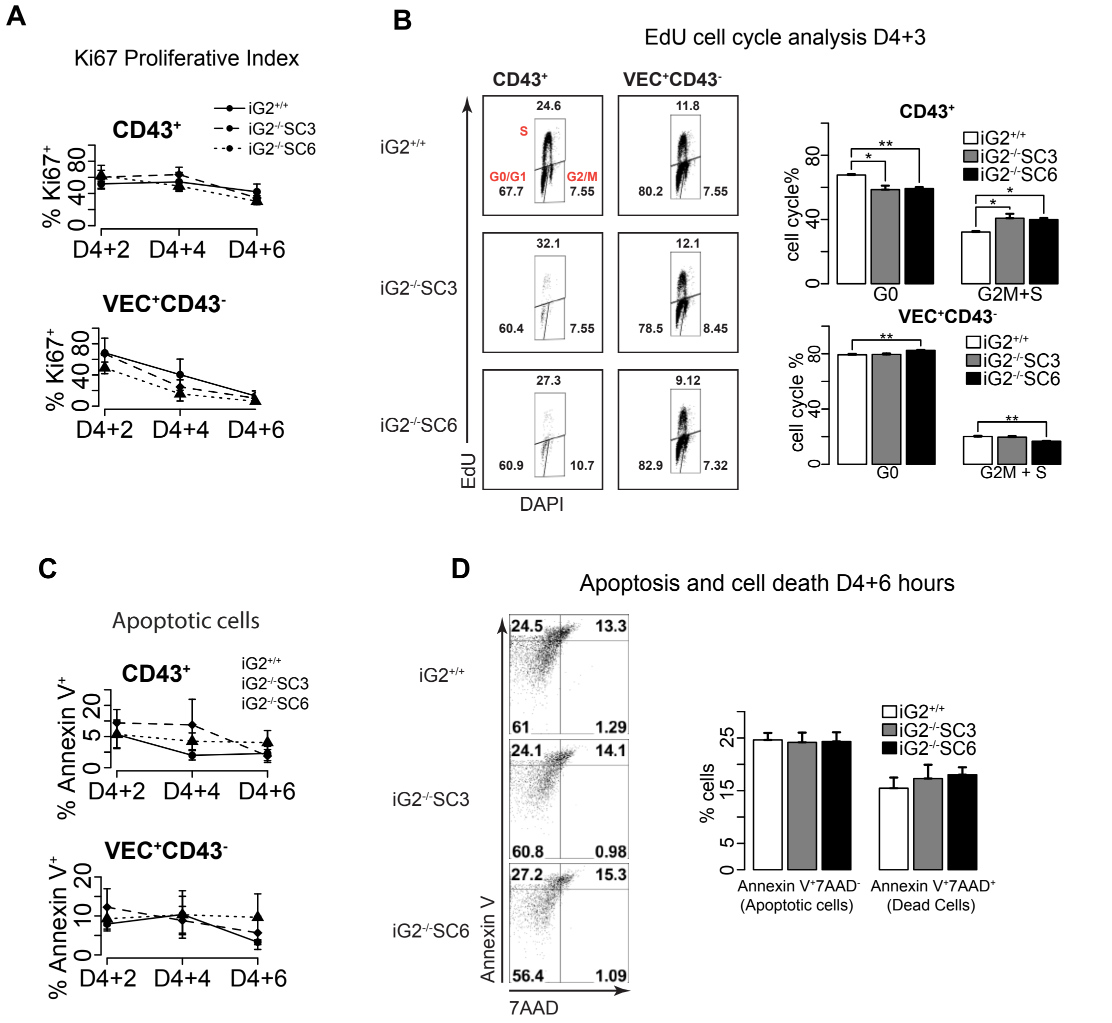

Figure S5. Analysis of proliferative potential and apoptosis in secondary cultures of D4 HE, related to Figure 5. (A) Analysis of cell proliferation in secondary cultures of day 4 (D4) HE using Ki67 immunostaining. Results are mean±SE for 3 independent experiments. (B) Representative dot plots and bar graphs show flow cytometric analysis of cell cycle using EdU and DAPI staining on day 4+3 HE culture. Bar graphs are mean±SE (n=3). (C) Assessment of apoptosis in secondary cultures of day 4 HE using annexin V flow cytometric analysis. (D) Representative dot plots and bar graphs show flow cytometric analysis of apoptosis and necrosis 6 hours post-plating of day 4 HE in secondary cultures. Bar graphs are mean±SE.

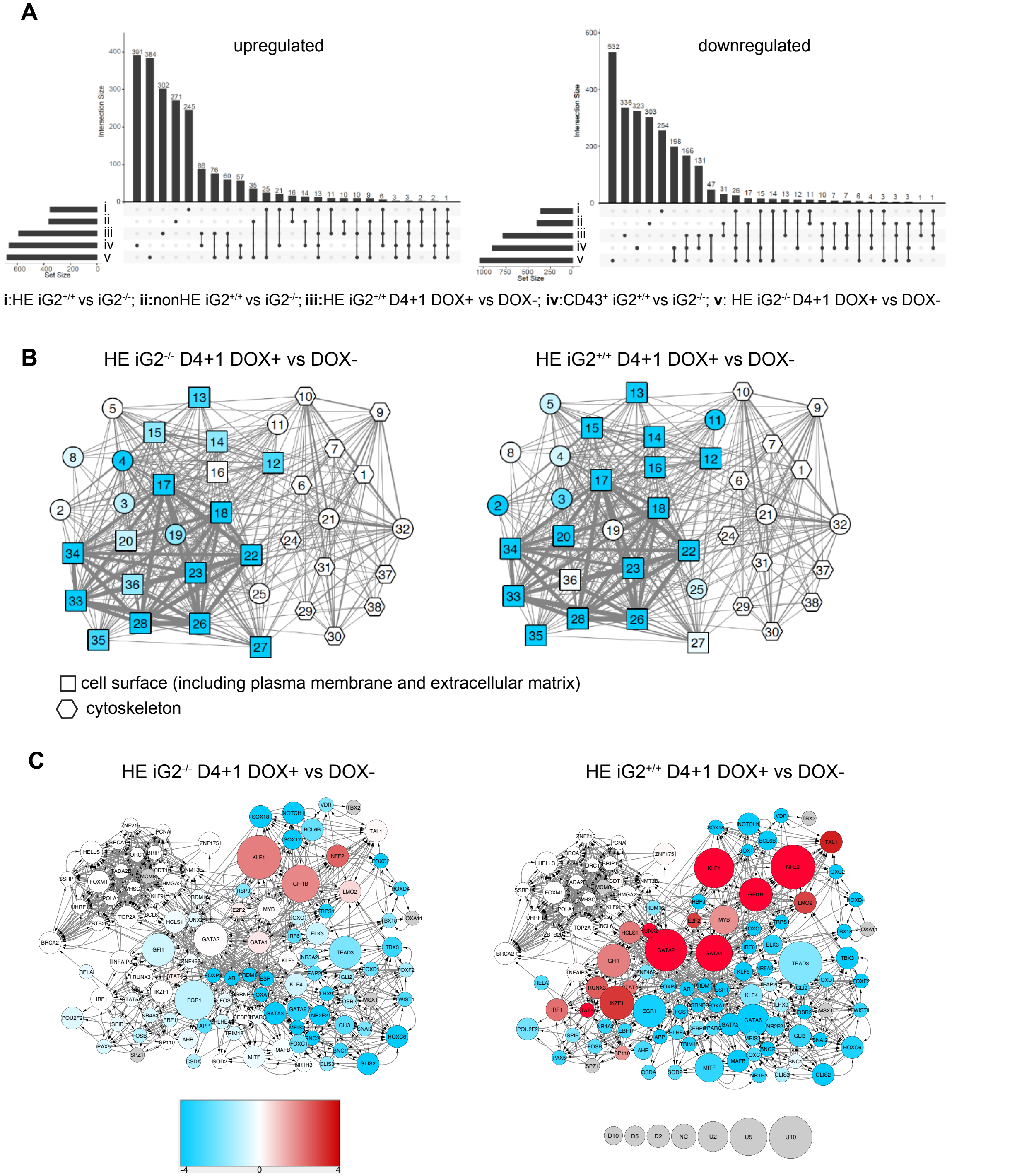

Figure S6. Gene expression profiling reveals distinct features of GATA2 regulatory network during hematopoietic development, related to Figure 6. (A) Bar chart illustrating multi-set intersections of genes upregulated (left chart) and downregulated (right chart) expressed in indicated cell subsets. The combination

matrix marked with circles identifies the genes uniquely upregulated by cell subset/subsets (intersections), while the overlying bars encode the set size and bars on the right show total number of genes uniquely upregulated. (B) GO analysis shows main GOCC categories (cell surface (including plasma membrane and extracellular matrix) and cytoskeleton) found to be affected by GATA2 in indicated cell subsets. Nodes representing those two supercategories are coded by shape, with squares representing cell surface and hexagons representing cytoskeleton. The color density represents enrichment (red) or depletion (blue) of differentially expressed genes related to displayed category. The width of the edges reflects the number of genes shared by categories. The "intracellular non membrane bound organelle" category (#21) that is clearly clustered with the cytoskeleton cohort, is not shape-coded since it does not formally fit the proposed supercategory definition (it encompasses chromosome, cytoskeleton and ribosomes). In our data, ribosome components are not responsive, however chromosome and cytoskeleton-related genes drive enrichment of this category to high significance levels. The node identification numbers correspond to the following GOCC categories: 1 - actin cytoskeleton, 2 - basal lamina, 3 - basement membrane, 4 - cell fraction, 5 - cell projection, 6 – centrosome, 7 - chromosomepericentric region, 8 – collagen, 9 - cytoskeletal part, 10 – cytoskeleton, 11 - dystrophin associated glycoprotein complex, 12 - extracellular matrix, 13 - extracellular matrix part, 14 - extracellular region, 15 - extracellular region part, 16 - extracellular space, 17 - integral to membrane, 18 - integral to plasma membrane, 19 - integrin complex, 20 - intercellular junction, 21 - intracellular non membrane bound organelle, 22 - intrinsic to membrane, 23 - intrinsic to plasma membrane, 24 - kinesin complex, 25 - leading edge, 26 – membrane, 27 - membrane fraction, 28 - membrane part, 29 - microtubule associated complex, 30 - microtubule cytoskeleton, 31 - microtubule organizing center, 32 - non membrane bound organelle, 33 - plasma membrane, 34 - plasma membrane part, 35 - proteinaceous extracellular matrix, 36 - receptor complex, 37 – spindle, 38 - spindle pole. (C) GATA2 transcriptional regulatory network reconstructed based on analysis of differentially expressed genes in $iG2^{+/+}$ and $iG2^{-/-}$ cells. Size of the nodes represents relative abundance of mRNA of the respective gene, computed as log_2(fold change) in $iG2^{+/+}$ versus $iG2^{-/-}$ cells. Both up- and downregulation effects are mapped onto the node size. The color density represents enrichment (red) or depletion (blue) of known targets of that transcription factor (regulon members) among the differentially expressed genes. Network visualization was performed using Cytoscape ver. 3.4.0.

Table S1. Expression of typical HE and non-HE-enriched genes in $iG2^{+/+}$ and $iG2^{-/-}$ HE and non-HE cells and $CD43^{+}$ HPs, Related to Figure 4.

Genes	$iG2^{+/+}$HE	$iG2^{-/-}$HE	$iG2^{+/+}$Non-HE	$iG2^{-/-}$Non-HE	$iG2^{+/+}$CD43	$iG2^{-/-}$CD43
RHAG	23.49	28.22	0.93	1.01	256.16	13.38
GFI1	89.36	65.5	2.64	1.16	10.83	6.38
RUNX1	24.26	11	7.31	3.56	57.57	62.17
NTS	377.13	149.51	32.95	21.22	95.89	17.25
BMPER	140.44	66.38	29.63	34.7	21.5	30.33
SOX17	5.36	21.15	76.98	59.46	0.19	22.59
COL15A1	3.24	6.37	96.78	94.76	2.11	158.94
CAV1	2.38	6.55	16.12	17.21	0.92	7.46
SCG5	0.26	0	14.98	21.27	1.86	1.74
NT5E	0.08	0.24	71.82	35.36	1.25	24.69
EMCN	0.49	1.24	42.37	32.88	0.45	12.76

Table S2. Genomic DNA-PCR for gRNA-mediated mutation screening Related to Experimental Procedures.

**Primers**	**Sequence (5' → 3')**	**Amplified region**	**Product Size (bp*)**
Forward	TGCTCTTTCTCGCCGGATCT	Exon 2	515
Reverse	CCCGCCCCAATTTTTCAGCA		
Forward	CCATGGTTCTGAGATGCTTTG	Exon 5	
Reverse	TCTTACGGGAAGCCCTTCTG		422
Reverse	TTCCCTAGTCCGAGACCCTG		483

*base pairs

Table S3. Primers for qRT-PCR, Related to Experimental Procedure

Genes	Primers	Sequence (5' → 3')	Product Size (bp)
GATA1	Forward	ACCTCCTGACCCTGGGACCT	307
	Reverse	TTCTTGGGCCGGATGAGGGG	
GATA2	Forward	GCGTCTCCTACAGCCCCGCGCAC	360
	Reverse	GGTGCTAGGGTCAGGAGACACTTC	
GATA3	Forward	CTGCCGGAGGAGGTGGATGTGC	248
	Reverse	GGACGTCTTGGAGAAGGGGCTGAG	
GATA4	Forward	GTGCCAACTGCCAGACCACCACC	341
	Reverse	CTGGGACACGGAGCTGCTGTGCC	
GATA5	Forward	TACCACAAGATGAATGGCGT	221
	Reverse	TTCCGTGTCTGGATGCTTTC	
GATA6	Forward	GAGGGAATTCAAACCAGGA	168
	Reverse	CCCTGAGGCTGTAGGTTGTGTTG	
RPL13a	Forward	CCACCCTGGAGGAGAAGAGG	138
	Reverse	CGTGGGTCTTGAGGACCTCTG	
GAPDH	Forward	TTCCAATATGATTCCACCCA	106
	Reverse	GATCTCGCTCCTGGAAGATG	
