# Supplementary material for: GATA2 Is Dispensable for Specification of Hemogenic Endothelium but Promotes Endothelial-to-Hematopoietic Transition
Source: Stem Cell Reports. 2018 May 31;11(1):197–211. doi: 10.1016/j.stemcr.2018.05.002 (PMC6066910; doi:10.1016/j.stemcr.2018.05.002)
Supplement: Document S2. Article plus Supplemental Information [file mmc3.pdf]

Stem Cell Reports
Article
OPEN ACCESS

GATA2 Is Dispensable for Specification of Hemogenic Endothelium but Promotes Endothelial-to-Hematopoietic Transition

HyunJun Kang,[1] Walatta-Tseyon Mesquitta,[1] Ho Sun Jung,[1] Oleg V. Moskvin,[1] James A. Thomson,[2,3,4] and Igor I. Slukvin[1,3,5,*]
[1]Wisconsin National Primate Research Center, University of Wisconsin Graduate School, 1220 Capitol Court, Madison, WI 53715, USA
[2]Morgridge Institute for Research, 330 N. Orchard Street, Madison, WI 53715, USA
[3]Department of Cell and Regenerative Biology, University of Wisconsin School of Medicine and Public Health, Madison, WI 53707-7365, USA
[4]Department of Molecular, Cellular, and Developmental Biology, University of California, Santa Barbara, CA 93106, USA
[5]Department of Pathology and Laboratory Medicine, University of Wisconsin Medical School, 600 Highland Avenue, Madison, WI 53792, USA
*Correspondence: islukvin@wisc.edu
https://doi.org/10.1016/j.stemcr.2018.05.002

SUMMARY

The transcriptional factor GATA2 is required for blood and hematopoietic stem cell formation during the hemogenic endothelium (HE) stage of development in the embryo. However, it is unclear if GATA2 controls HE lineage specification or if it solely regulates endothelial-to-hematopoietic transition (EHT). To address this problem, we innovated a unique system, which involved generating *GATA2* knockout human embryonic stem cell (hESC) lines with conditional GATA2 expression (iG2$^{-/-}$ hESCs). We demonstrated that GATA2 activity is not required for VE-cadherin^{+}CD43^{-}CD73^{+} non-HE or VE-cadherin^{+}CD43^{-}CD73^{-} HE generation and subsequent HE diversification into DLL4^{+} arterial and DLL4^{-} non-arterial lineages. However, GATA2 is primarily needed for HE to undergo EHT. Forced expression of GATA2 in non-HE failed to induce blood formation. The lack of GATA2 requirement for generation of HE and non-HE indicates the critical role of GATA2-independent pathways in specification of these two distinct endothelial lineages.

INTRODUCTION

The formation of blood cells from hemogenic endothelium (HE) is a key element of embryogenesis leading to establishment of the hematopoietic system. It has become increasingly clear that HE represents a distinct subset of RUNX1-expressing CD73^{-} vascular endothelium capable of undergoing endothelial-to-hematopoietic transition (EHT) (Choi et al., 2012; Ditadi et al., 2015; Jaffredo et al., 2010; North et al., 1999; Slukvin, 2016) and that hematopoietic specification occurs at the HE stage (Elcheva et al., 2014; Guibentif et al., 2017). However, the mechanisms guiding EHT and specification of HE lineage are poorly understood. A number of transcription factors including RUNX1, GATA2, GFI1, HOXA3, SOX17, and TAL1, and NOTCH, WNT, and BMP/TGF-β signaling have been implicated in control of HE and blood development (reviewed in Slukvin, 2016; Swiers et al., 2013b; Thambyrajah et al., 2016b). GATA2 transcription factor is of particular interest since it is critical for development of the entire hematopoietic system, including hematopoietic stem cells (HSCs) during embryogenesis. GATA2 deficiency in mice leads to early embryonic lethality (E10–E10.5), and markedly impaired primitive yolk sac and definitive embryonic hematopoiesis (Tsai et al., 1994). GATA2 deficiency also impairs hematopoiesis in mouse and human pluripotent stem cells (hPSC) cultures (Huang et al., 2015; Tsai and Orkin, 1997). Overexpression of GATA2 along with ETV2 or TAL1 in hPSCs directly induces HE with pan-myeloid or erythro-megakaryocytic potentials (Elcheva et al., 2014).

Conditional knockout of *GATA2* in VE-cadherin (VEC)-expressing endothelial cells, along with analysis of aorta-gonad-mesonephros (AGM) hematopoiesis in mice with deleted *Gata2* +9.5 *cis*-element, revealed that GATA2 is required for the formation of intra-aortic hematopoietic clusters and HSCs (de Pater et al., 2013; Eich et al., 2018; Gao et al., 2013; Lim et al., 2012). The effect of GATA2 at this stage can be attributed to two mechanisms: (1) GATA2 selectively abrogates generation of HE lineage, and therefore hematopoiesis, but has no effect on non-HE or (2) GATA2 does not affect HE specification, but rather promotes EHT. It is also possible, that GATA2 may affect both mechanisms, or act in cell-non-autonomous manner, by mediating environmental signaling to HE from non-HE.

To provide mechanistic insights on the exact role of GATA2 in blood development during the EHT, we developed a unique GATA2-dependent hematopoietic rescue system. This system was comprised of a doxycycline (DOX)-inducible GATA2 hESC line, in which endogenous *GATA2* had been knocked out. This enabled us to probe the effect of GATA2 at distinct stages of hematopoiesis. We demonstrated that GATA2 is not required for non-HE and HE specification, or HE diversification into arterial and non-arterial HE, which suggests that these developmental stages are predominantly regulated by GATA2-independent mechanisms. GATA2 rescued in HE restored EHT and blood formation. In contrast to HE, enforced expression of GATA2 in non-HE fails to induce substantial EHT and blood production. Reconstruction of the GATA2 network based on publicly available regulatory interactions

This is an open access article under the CC BY license (http://creativecommons.org/licenses/by/4.0/).

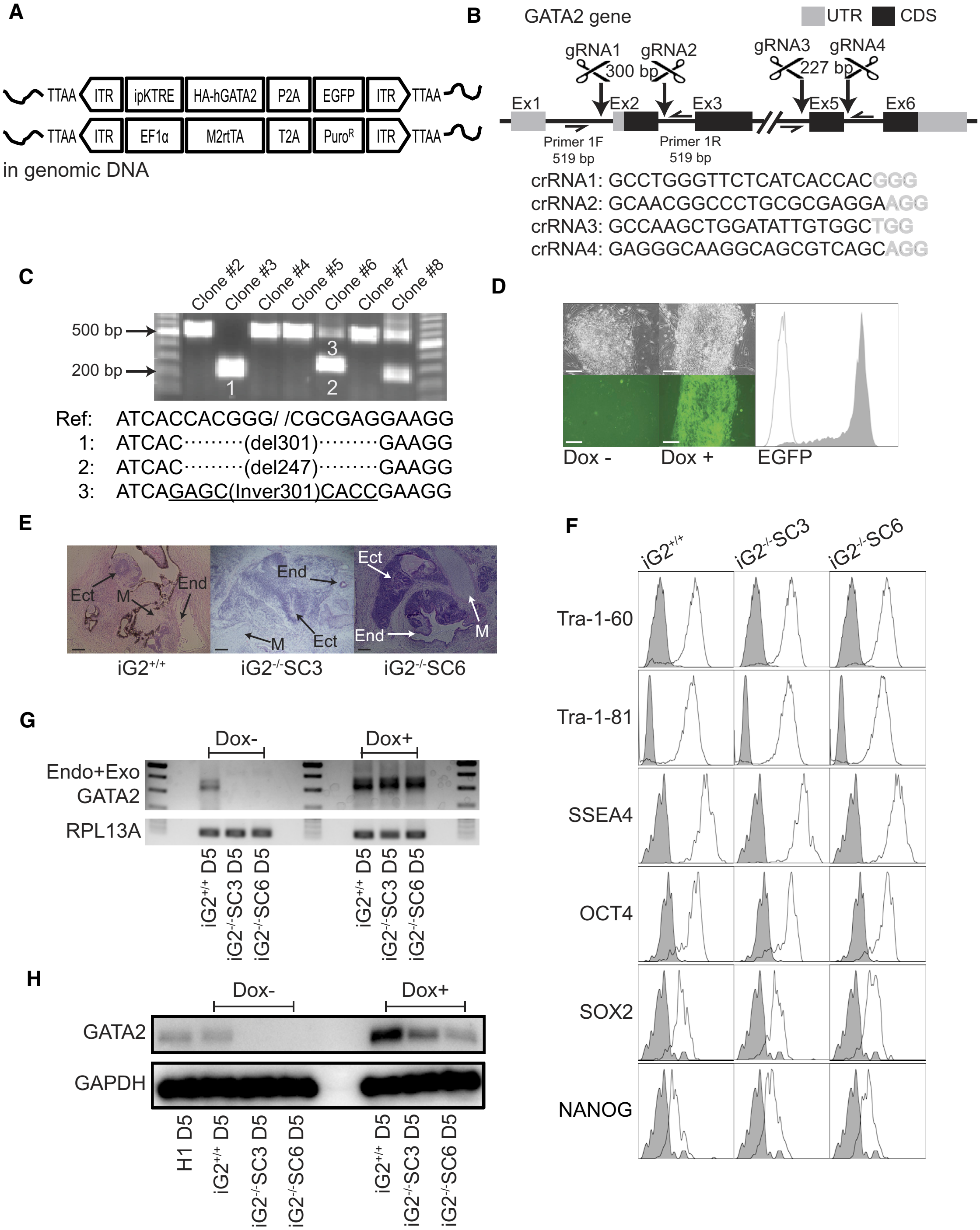

Figure 1. Generating GATA2 DOX-Inducible hESC Lines with Endogenous GATA2 Knockout

(A) Schematic illustration of PiggyBac system used to generate GATA2 DOX-inducible (iG2$^{+/+}$) hESCs.

(B) Strategy for GATA2 knockout in iG2$^{+/+}$ hESCs. Two pairs of guide RNAs (gRNAs) designed to target exons 2 and 5, respectively. Nucleotides in gray are the protospacer adjacent motif sequences known as "NGG."

(C) PCR amplification with genomic DNA extracted from each clone recovered from single-cell sorting of gRNAs and Cas9-transfected cells. Sequencing of amplicons from genomic DNA-PCR shows deletion and/or conversion of a large *GATA2* fragments: clone no. 3 (iG2$^{-/-}$SC3) has biallelic 301 bp deletion, and clone no. 6 (iG2$^{-/-}$SC6) has 247 bp deletion in one allele and a 301 bp inversion in the other allele in the intron-exon 2-intron *GATA2* coding region.

(legend continued on next page)

and our molecular profiling of wild-type and GATA2-deficient cells, suggested distinct GATA2-dependent molecular programs operating in HE and non-HE, and that mechanisms upstream of GATA2, are most critical for establishing HE. In addition, we showed that GATA2-deficient cells are still able to produce a limited number of GATA2-independent hematopoietic progenitors (HPs), albeit with markedly reduced erythroid and granulocytic potentials, but retaining macrophage, T, and natural killer (NK) lymphoid cells.

RESULTS

Generation of GATA2 Conditional and Knockout hESC Lines

To study GATA2 function during hematopoietic development, we engineered an H1 human embryonic stem cell (hESC) line carrying a DOX-inducible *GATA2* transgene with a modified tetracycline response element (ipKTRE) that was designed to enhance resistance to transgene silencing (Figure S1A), using the PiggyBac transposon system (Figure 1A; iG2$^{+/+}$ hESCs). The CRISPR/Cas9 system was then used to knockout endogenous *GATA2* with targeted guide RNA sequences around exons 2 and 5 (Figure 1B). Following single-cell cloning, we established two clonal cell lines (iG2$^{-/-}$SC3 and iG2$^{-/-}$SC6). One with a biallelic 301 bp deletion in the coding region (iG2$^{-/-}$SC3), and the other one with a 247 bp deletion in one allele, and a 301 bp inversion in the other allele in the intron-exon 2-intron coding region (iG2$^{-/-}$SC6) (Figure 1C). These mutations removed the translation initiation codon and transactivation domain and introduced a premature stop codon. However, no genomic alterations were observed in the second targeted genomic region around exon 5 (Figure S1B). All genetically engineered H1 cell lines maintained typical hESC morphology (Figure 1D), formed teratomas with three germ layers in immunodeficient mice (Figure 1E), and expressed pluripotency genes (Figure 1F). To evaluate GATA2 expression, we differentiated wild-type H1 and engineered hESC lines in chemically defined conditions for 5 days to induce formation of hematoendothelial progenitors, in which endogenous GATA2 expression is substantially upregulated according to our previous expression profiling (Choi et al., 2012; Uenishi et al., 2014), and assessed GATA2 expression by qRT-PCR and western blot. As shown in Figures 1G, 1H, S2A, and S2B, wild-type H1 and iG2$^{+/+}$H1 hESC lines maintained endogenous GATA2 expression. No endogenous or exogenous GATA2 expression was observed in the two iG2$^{-/-}$H1 hESC lines without DOX, and GATA2 upregulation was confirmed following DOX treatment. In control cultures with wild-type H1 hESCs, DOX did not affect GATA2 expression (Figure S2A) or hematopoietic differentiation (Figure S2C). Thus, generated hESC lines allow for precise modulation of GATA2 expression in the setting of intact or genomic *GATA2* knockout.

GATA2 Deficiency Severely Impairs hESC Differentiation into HPs

To determine whether the effect of GATA2 on blood development in humans is similar to that observed in the mouse embryo, we performed hematopoietic differentiation of iG2$^{-/-}$H1 cell lines in chemically defined conditions (Uenishi et al., 2014). In this differentiation system, hESCs undergo stepwise progression into APLNR^{+}PDGFRα^{+} primitive posterior mesoderm with hemangioblast colony-forming cells (HB-CFCs) that reflects primitive hematopoiesis, KDR^{hi}PDGFRα$^{lo/-}$VEC^{-} hematovascular mesodermal progenitors with definitive hematopoietic potential; immature VEC^{+}CD43^{-}CD73^{-} HE, which specify into DLL4^{+} arterial HE with definitive hematopoietic potential and DLL4^{-} non-arterial-type HE with mostly primitive hematopoietic potential; and finally CD43^{+} HPs that include CD235^{+}CD41^{+}CD45$^{-/+}$ erythromegakaryocytic progenitors (E-MkP) and CD235/41^{-}CD45$^{+/-}$ multipotent HPs (MHPs) with a lin^{-}CD34^{+}CD90^{+}CD38^{-}CD45RA^{-} hematopoietic stem progenitor cell phenotype (Choi et al., 2009a, 2009b, 2012; Uenishi et al., 2018; Vodyanik et al., 2006) (Figure 2A). As shown in Figures 2B and 2C, loss of GATA2 was associated with a significant reduction in HB-CFCs on day 3 of differentiation, without change in cellular composition of HB colonies. As determined by flow cytometry, iG2$^{+/+}$ and iG2$^{-/-}$ HB colonies collected from day 12 clonogenic cultures were composed predominantly of

(D) Microscopic and flow cytometric examination of transgene expression. EGFP signal under DOX treatment reporting expression of GATA2. Scale bars, 100 μm.
(E) Teratoma formation to evaluate pluripotency of genetically modified hESCs. Derivatives of three germ layers are recognized: Ect, ectoderm; M, mesoderm; End, endoderm. Scale bar, 200 μm.
(F) Surface and intracellular pluripotency markers were confirmed by flow cytometry. Plots depict isotype control (gray) and specific antibody (open) histograms.
(G) qRT-PCR analysis of GATA2 expression in iG2$^{-/-}$ and iG2$^{+/+}$ day 5 differentiated cells.
(H) Western blot with proteins extracted at day 5 of differentiation, confirming the absence of GATA2 expression in GATA2 knockout cells and induction of GATA2 following DOX treatment.
See also Figures S1 and S2.

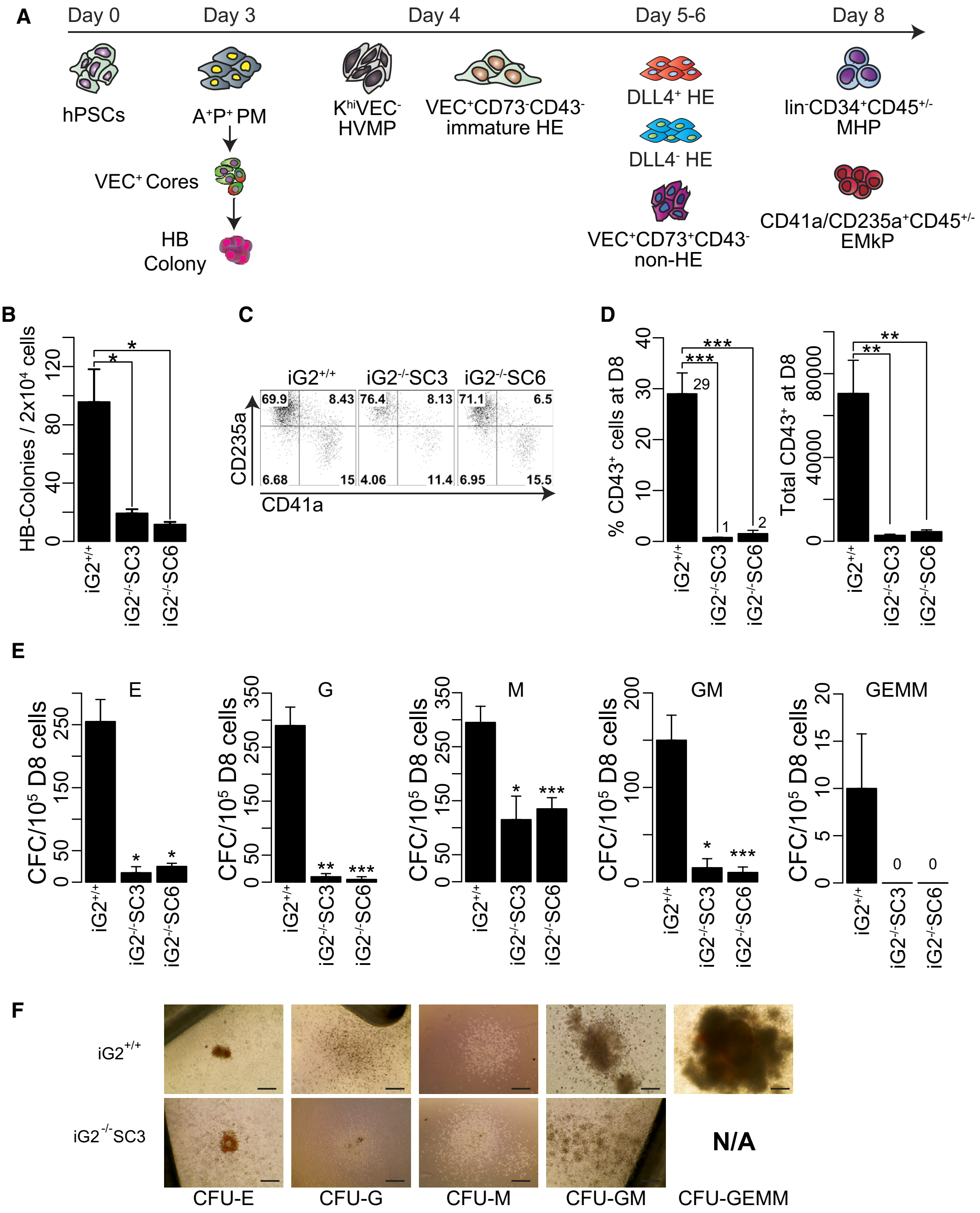

Figure 2. GATA2 Deficiency Significantly Impairs Hematopoietic Development of hPSCs

(A) Schematic diagram depicts major stages of hematopoietic development and cell populations analyzed in hESC differentiation cultures. A^+P^+ PM, $APLNR^+PDGFR\alpha^+$ primitive posterior mesoderm; HB, hemangioblast; $K^{hi}VEC^-$ HVMPs, $KDR^{high}PDGFR\alpha^{low/-}VEC^-$ hematovascular mesodermal progenitors; HE, hemogenic endothelium; MHPs, multipotent hematopoietic progenitors; EMkPs, erythromegakaryocytic progenitors.

(B) Frequency of HB colonies.

(C) Flow cytometric analysis of the hematopoietic composition of HB colonies. Representative dot plots of CD43-gated cells collected from clonogenic cultures are shown.

(legend continued on next page)

CD235a$^+$ and CD41$^+$ erythroid and megakaryocytic lineage cells, similar to our prior findings with wild-type hPSCs (Choi et al., 2012). In addition, analysis of blood formation on day 8 of iG2$^{-/-}$ hESC differentiation revealed a profound (approximately 30-fold) reduction in CD43$^+$ HPs compared with iG2$^{+/+}$ cells (Figure 2D). In a colony-forming assay, iG2$^{-/-}$ cultures generated far less total CFCs compared with iG2$^{+/+}$ cells, with all types of CFCs experiencing a significant reduction (Figure 2E). However, in contrast to mouse studies (Kaimakis et al., 2016; Tsai and Orkin, 1997), we did not observe marked differences in the size of hematopoietic colonies between hESCs with intact and knockout *GATA2* (Figure 2F), which is consistent with prior observations in human *GATA2*-knockout hESCs (Huang et al., 2015). Thus, we concluded that GATA2 deficiency significantly impairs hematopoiesis from hESCs, and that the iG2$^{-/-}$ hESC differentiation system is suitable for assessing conditional rescue of GATA2 expression on hematopoietic development.

GATA2-Independent HPs Have Reduced Granulocytic Potential but Are Competent to Differentiate into Macrophage, T, and NK Lymphoid Cells

In mice, the absence of GATA2 does not completely ablate hematopoiesis in the embryo (de Pater et al., 2013; Tsai et al., 1994; Tsai and Orkin, 1997), and GATA2-independent HPs have been recently described (Canete et al., 2017; Kaimakis et al., 2016). Similar to mouse, we observed the production of a very small number of hematopoietic cells in the absence of GATA2 expression in the human system (Figures 2D and 3A). To characterize these GATA2-independent progenitors, we analyzed phenotype and function of the CD43$^+$ cells isolated from iG2$^{-/-}$ and iG2$^{+/+}$ hESCs. As shown in Figure 3B, all typical CD43$^+$ subsets (E-MkPs and MHPs) described in wild-type hESCs were present in cultures from GATA2-ablated hESCs. However, we observed a relative decrease in CD235/CD41a$^+$ E-MkPs, especially in the CD235a/CD41a$^+$CD45$^+$ E-MkP subset, with a relative increase in CD235/CD41$^-$CD45$^-$ MHPs from iG2$^{-/-}$ cells. Analysis of CFC potential of isolated CD43$^+$ cells revealed that, when compared with iG2$^{+/+}$ cells, iG2$^{-/-}$ CD43$^+$ cells produced substantially less CFC-GM, CFC-G, and CFC-E, but exhibited no differences in CFC-M (Figure 3C). When iG2$^{-/-}$ CD43$^+$ cells were cultured in lymphoid conditions on DLL4-OP9, they produced T and NK cells in quantities similar to iG2$^{+/+}$ CD43$^+$ cells (Figures 3D–3G). In mouse, Gata2-independent HPs are likely supported through the function of Gata3 and Gata4 (Canete et al., 2017; Kaimakis et al., 2016). To exploit whether this is true for hESC-generated progenitors, we analyzed expression of these GATA factors in CD43$^+$ cells. As shown in Figure S3, CD43$^+$ cells from iG2$^{-/-}$ hESCs showed elevated expression of *GATA3, GATA4, GATA5*, and *GATA6* genes, thereby suggesting that CD43$^+$ cells generated from iG2$^{-/-}$ hESCs may be similar to Gata2-independent HPs described in the mouse system.

GATA2 Is Dispensable for Development of HE and Its Arterial Specification

To define GATA2-dependent steps in hematopoiesis, we treated iG2$^{-/-}$ and iG2$^{+/+}$ hESCs with DOX in a stepwise manner, as depicted in Figure 4A. As shown in Figures 4B–4E, DOX treatment of iG2$^{-/-}$ and iG2$^{+/+}$ has the greatest effect on CD43$^+$ cell production and CFC potential when performed on days 3–4 or 4–5 of differentiation. In contrast, DOX treatment on days 0–2 suppressed differentiation, while treatment on days 5–6 showed little effect. Since formation of HE and EHT in our system occurs during days 4–5 of differentiation (Choi et al., 2012; Uenishi et al., 2014), i.e., when we see the most dramatic effect of DOX treatment, we concluded that GATA2 may be important for HE formation or EHT. To define the effect of GATA2 at EHT stage more precisely, we evaluated major mesodermal subsets and HE in iG2$^{-/-}$ and iG2$^{+/+}$ cultures by flow cytometry. As shown in Figure 4F, the absence of GATA2 has little effect on APLNR$^+$PDGFRα^+ primitive posterior mesoderm (day 3), which possesses the potential to form HB colonies through endothelial intermediates in semi-solid medium in response to fibroblast growth factor 2 (FGF-2) (Choi et al., 2012; Vodyanik et al., 2010). GATA2 deficiency also had minimal effect on formation of KDR^{hi}VEC$^-$ hematovascular mesodermal precursors or immature VEC$^+$CD43$^-$CD73$^-$ HE on day 4 of differentiation (Figure 4G). Analysis of the VEC$^+$ cell subset on day 5 of differentiation revealed that endothelial cells with VEC$^+$CD43$^-$CD73$^-$ HE and VEC$^+$CD43$^-$CD73$^+$ non-HE phenotypes are formed in iG2$^{-/-}$ cultures, although we observed a slight increase in phenotypical HE and a significant increase in non-HE from iG2$^{-/-}$ compared with iG2$^{+/+}$ differentiation cultures (Figure 4H). In previous studies, we defined a set of markers to distinguish HE and non-HE (Choi et al., 2012). Characteristically, HE cells express higher levels of *RHAG, GFI1, RUNX1, NTS*, and *BMPER* genes, while non-HE cells express higher levels of *SOX17, COL15A1, CAV1, SCG5,* and *EMCN* genes. As determined

(D) Percentage and absolute number of CD43$^+$ generated from iG2$^{+/+}$ and iG2$^{-/-}$ hESCs on day 8 of differentiation.
(E) Hematopoietic CFC potential on day 8 of differentiation.
(F) Representative images of hematopoietic colony-forming units (CFUs). Scale bar, 100 μm.
Bars in (B)–(D) are means ± SE for at least three independent experiments. *p < 0.05, **p < 0.01, ***p < 0.001.

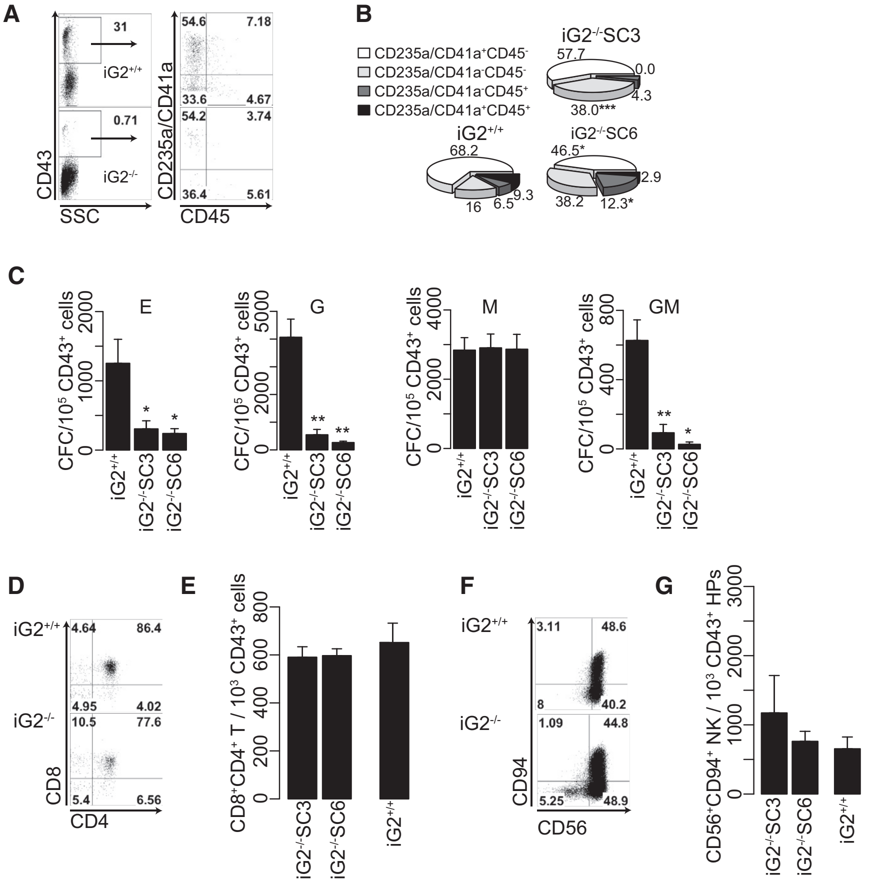

Figure 3. Characterization of iG2$^{-/-}$ CD43^{+} HPs

(A) Flow cytometry dot plots comparing CD43^{+} subsets in iG2$^{+/+}$ and iG2$^{-/-}$ cultures on day 8 of differentiation.

(B) Phi chart depicting the mean percentage of each CD43^{+} subset for three independent experiments.

(C) CFC potential of magnetic-activated cell sorting-purified iG2$^{+/+}$ iG2$^{-/-}$ CD43^{+} cells isolated on day 8 of differentiation.

(D) Representative flow cytometry dot plots displaying T cell differentiation from iG2$^{+/+}$ and iG2$^{-/-}$CD43^{+} cells.

(E) Absolute number of CD8^{+}CD4^{+} T cell progenitors generated from 1,000 of iG2$^{+/+}$ and iG2$^{-/-}$ CD43^{+} cells.

(F) Representative flow cytometry dot plots displaying NK cell differentiation from iG2$^{+/+}$ and iG2$^{-/-}$CD43^{+} cells.

(G) Absolute number of CD8^{+}CD4^{+} T cell progenitors generated from 1,000 iG2$^{+/+}$ and iG2$^{-/-}$ CD43^{+} cells.

Bars in (C), (E), and (G) are means ± SE for at least three independent experiments. *$p < 0.05$, **$p < 0.01$, ***$p < 0.001$. See also Figure S3.

by RNA sequencing (RNA-seq) analysis, the aforementioned pattern of marker distribution in iG2$^{+/+}$ and iG2$^{-/-}$ HE and non-HE was similar, i.e., higher expression of *RHAG, GFI1, RUNX1, NTS*, and *BMPER* genes in HE, while higher expression of *SOX17, COL15A1, CAV1, SCG5*, and *EMCN* was found in non-HE (Figure 4I; Table S1), thereby confirming that the VEC^{+}CD43^{-}CD73^{-} and VEC^{+}CD43^{-}CD73^{+} phenotypes in iG2$^{-/-}$ cells reliably separate HE from non-HE. However, we noticed downregulation of *RUNX1, NTS*, and *BMPER* HE-enriched genes in iG2$^{-/-}$ HE cells compared with iG2$^{+/+}$ HE.

Recently, we revealed that expression of DLL4 within day 5 VEC^{+}CD43^{-}CD73^{-} HE defines arterial-type HE which is highly enriched in definitive HPs, while DLL4^{-} non-arterial HE produces cells with primitive hematopoietic potential (Uenishi et al., 2018). Analysis of DLL4 expression in iG2$^{-/-}$ and iG2$^{+/+}$ HE, demonstrated that the absence of GATA2 does not abrogate specification of DLL4^{+} and DLL4^{-} HE subsets (Figure 4H), thus suggesting that, despite the dramatic effect of GATA2 on hematopoietic cells, it is dispensable for HE and non-HE specification, and subsequent HE diversification into DLL4^{+} arterial and DLL4^{-} non-arterial lineages.

GATA2 Regulates Blood Formation Primarily through Promotion of EHT

To establish whether GATA2 affects formation of hematopoietic cells primarily through EHT regulation, we assessed the effect of DOX treatment on HB colony development. As we demonstrated previously, HB colonies are composed of primitive hematopoietic cells that develop through primitive HE intermediates (cores) (Choi et al., 2012; Lancrin et al., 2009; Vodyanik et al., 2010). As shown in Figures 5A and 5B, iG2$^{-/-}$ cells isolated on day 3 of differentiation retained their capacity to form cores, although we observed an approximately 1.5-fold reduction in core numbers in iG2$^{-/-}$ cells compared with iG2$^{+/+}$ cells. Inducing GATA2 by adding DOX to clonogenic medium increased the number of cores in iG2$^{-/-}$ and iG2$^{+/+}$ cultures. Importantly, rescuing GATA2 expression restored transition of HE cores into primitive blood cells and led to development of mature HB colonies by iG2$^{-/-}$ cells (Figure 5B). Following DOX addition, we observed a more than 10-fold increase in HB colonies and a restoration in the HB colony/core ratio in iG2$^{-/-}$ cells, thereby indicating that GATA2 is required for transition of primitive HE to hematopoietic stage of development. Next, we isolated iG2$^{-/-}$ CD31^{+} endothelial cells

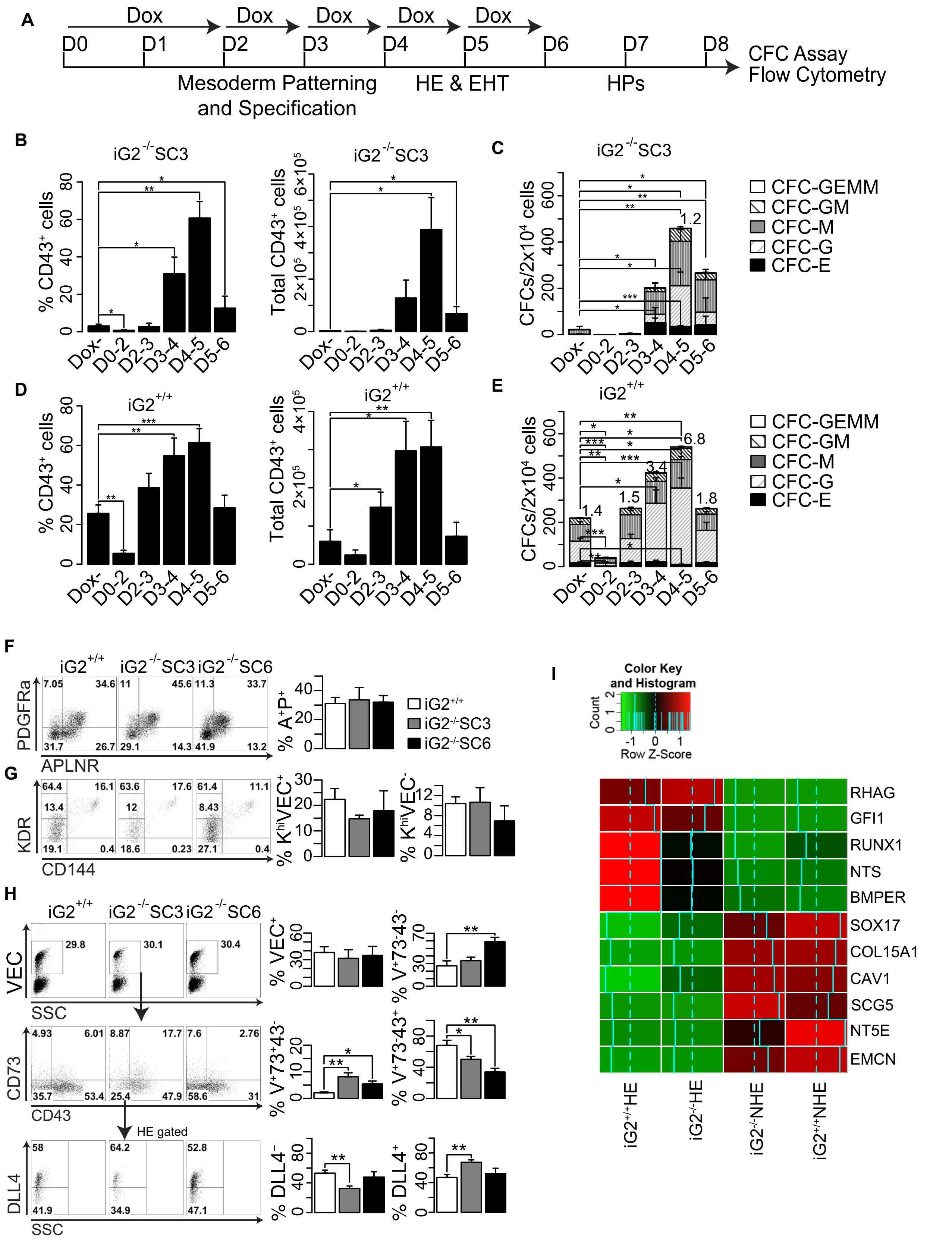

(legend on next page)

from day 4 of differentiation and assessed how DOX treatment affects blood formation from HE (Figure 5C). CD31^{+} cells on day 4 of differentiation represent a population of immature VEC^{+} HE lacking CD43 and CD73 expression (Choi et al., 2012; Uenishi et al., 2014). In the absence of DOX, iG2$^{-/-}$ HE produced very few blood cells, while DOX treatment restored HE capacity to form CD43^{+} hematopoietic cells and CFCs (Figures 5D–5G). In addition, DOX treatment enhanced CFC formation from iG2$^{+/+}$ cells (Figure 5G).

To further characterize the effect of GATA2, we performed clonal analysis of day 4 HE using the OP9 stromal cells and serum-containing medium, which supports hematoendothelial development from single cells (Choi et al., 2012). As shown in Figure 5H, iG2$^{-/-}$ cells demonstrated a much lower ratio of hematopoietic/endothelial colonies compared with iG2$^{+/+}$ cells. DOX treatment restored the formation of hematopoietic colonies and increased hematopoietic/endothelial ratio by more than 3-fold.

Recently, we demonstrated that day 4 immature HE progenitors undergo further specification into two subsets: DLL4^{+} arterial HE, which is enriched in definitive HPs and requires NOTCH signaling for EHT, and DLL4^{-} nonarterial HE with predominantly primitive hematopoietic potential (Uenishi et al., 2018). To assess whether GATA2 affects EHT from both types of HE, we isolated DLL4^{+} and DLL4^{-} HE from iG2$^{+/+}$ and iG2$^{-/-}$ cells on day 5 of differentiation, and cultured these subsets on DLL4-OP9 cells. As shown in Figures S4A and S4B, GATA2 deficiency affected EHT from both types of HE, which was consistent with the essential role of GATA2 in both primitive and definitive hematopoiesis.

The effect of GATA2 was specific to HE. When we isolated iG2$^{+/+}$ and iG2$^{-/-}$ VEC^{+}CD43^{-}CD73^{+} non-HE and cultured in HE conditions, very few blood cells were formed. Adding DOX had a negligible effect on blood production in these cultures (Figures S4C and S4D), thereby suggesting that GATA2 upregulation is not able to induce the hemogenic program and EHT in non-HE.

To establish whether GATA2 contributes specifically to EHT per se, or to proliferation and survival of CD43^{+} cells at post-EHT, we evaluated the potential effect of GATA2 on the proliferation and apoptosis of CD43^{+} cells emerging from HE at different time points during secondary differentiation. No significant differences were found in Ki67 proliferative indices between iG2$^{-/-}$ and iG2$^{+/+}$ CD43^{+} HPs or VEC^{+}CD43^{-} endothelial cells throughout days 4 + 1 to days 4 + 6 secondary differentiation (Figure S5A). Cell-cycle analysis performed on day 4 + 3 of differentiation using 5-ethynyl-2′-deoxyuridine, revealed a mild decrease in quiescent G0 and increase in proliferating (G2M + S) cells in iG2$^{-/-}$ cells compared with iG2$^{+/+}$ cells (Figure S5B). Thus, we concluded that decreased CD43^{+} cell proliferation cannot explain the impaired generation of blood cells from iG2$^{-/-}$ HE cells. As determined by annexin V staining, GATA2 deficiency did not affect survival of HPs and endothelial cells in secondary cultures of day 4 HE (Figure S5C). To exclude the possibility that GATA2 deficiency can cause a rapid death of iG2$^{-/-}$ HE cells in secondary cultures, we assessed apoptosis and cell death 6 hr after initiation of secondary culture. As shown in Figure S5D, no significant differences were found in apoptotic and necrotic cells between iG2$^{-/-}$ and iG2$^{+/+}$ cells. Altogether these findings imply a specific effect of GATA2 on EHT, rather than on apoptosis or proliferation of blood cells.

Analysis of GATA factors by qPCR revealed that, despite higher expression of *GATA3, GATA4, GATA5*, and *GATA6* factors in CD43^{+} HPs and non-HE from iG2$^{-/-}$ cells compared with iG2$^{+/+}$ cells, expression of these GATA factors was lower in iG2$^{-/-}$ HE compared with iG2$^{+/+}$ HE (Figure S3). These findings can be explained by the low frequencies of GATA2-independent HE cells within VEC^{+}CD43^{-}CD73^{-} population and by activation of GATA2-independent mechanisms at EHT and/or post-EHT stage.

Figure 4. Stage-Specific Effect of GATA2 on Hematopoietic Differentiation from hPSCs
(A) Schematic diagram of experiments to study the stage-wise effect of GATA2 on hematopoietic development.
(B and C) Percentage and absolute number of CD43^{+} blood cells (B) and CFC numbers (C) in day 8 iG2$^{-/-}$ differentiation cultures following stepwise DOX treatment. Bars are means ± SE for three independent experiments. *p <0.05, **p < 0.01, ***p < 0.001.
(D and E) Percentage and absolute number of CD43^{+} blood cells (D) and CFC numbers (E) in day 8 iG2$^{+/+}$ differentiation cultures following stepwise DOX treatment. (C and D) Bars are mean ± SE for seven independent experiments. *p <0.05, **p < 0.01, ***p < 0.001. Numbers on top show average CFC-GEMM frequencies.
(F–H) Formation of A^{+}P^{+} mesoderm (F), HVMP (G), and HE (VEC^{+}CD43^{-}CD73^{-}), and non-HE (VEC^{+}CD43^{-}CD73^{+}) and DLL4^{+} arterial-type population within HE (H) from iG2$^{+/+}$ and iG2$^{-/-}$ hESCs. (F and H) Bars are means ± SE for at least three independent experiments. *p <0.05, **p < 0.01.
(I) Heatmap showing expression of typical HE and non-HE-enriched genes in iG2$^{+/+}$ and iG2$^{-/-}$ VEC^{+}CD43^{-}CD73^{-} HE and VEC^{+}CD43^{-}CD73^{+} non-HE cells. Scaled gene expression, denoted as the row *z* scores, is displayed in a red-green color scale where red indicates high expression and green indicates low expression.
See also Table S1.

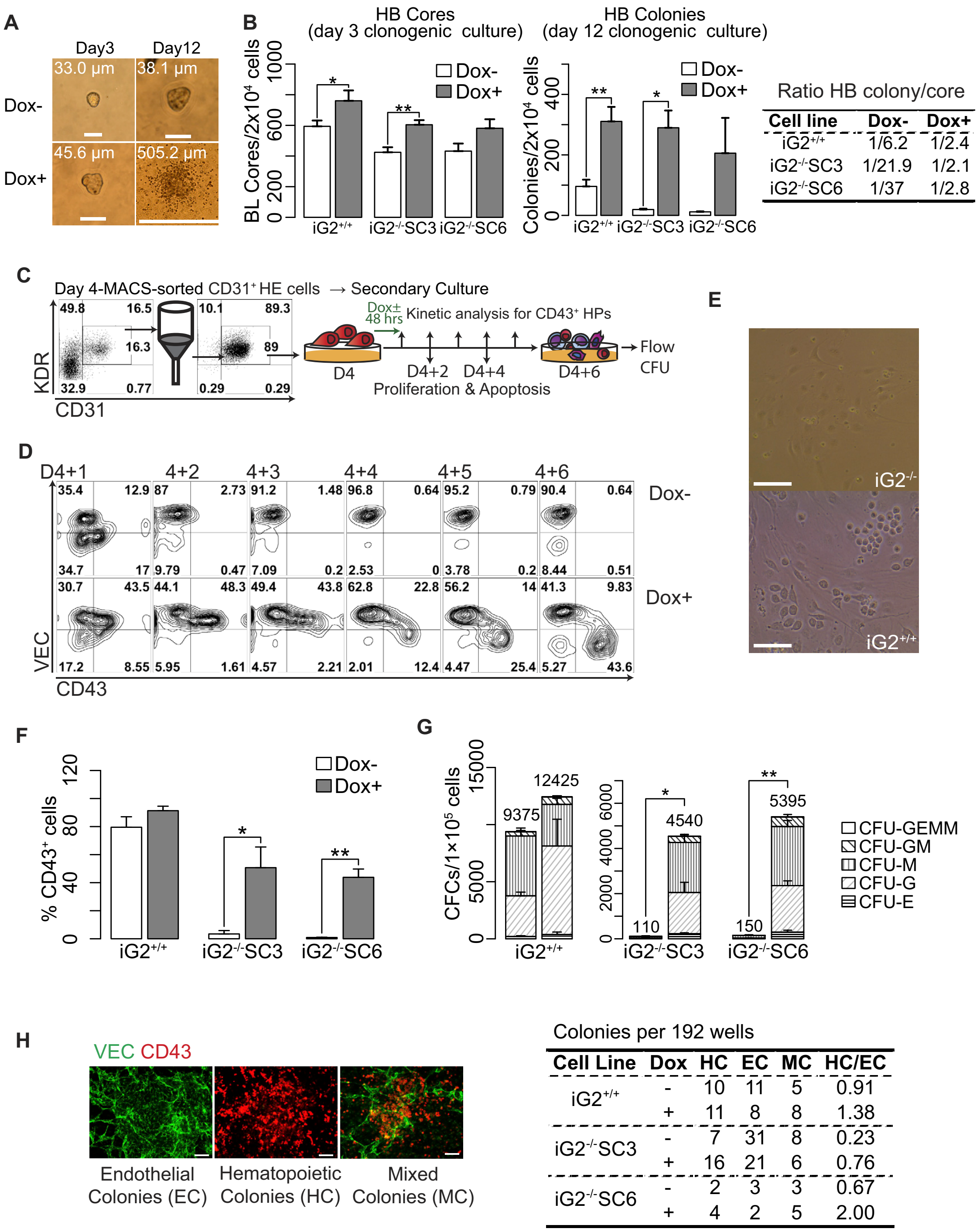

Figure 5. The Regulation of EHT by GATA2

(A) Microscopic images of HB colonies at day 3 and 12 of clonogenic cultures. In clonogenic cultures, mesodermal cells from iG2$^{-/-}$ cells form cores composed of endothelial intermediates that fail to undergo EHT and form dispersed HB colonies composed of hematopoietic cells.

(B) Frequencies of HB cores and HB colonies in clonogenic cultures with and without DOX.

(C) Schematic diagram of research strategy used to evaluate the effect of GATA2 on EHT. H&E cells were isolated from day 4 differentiation cultures (D4 HE) and cultured with DOX added during the first 48 hr or without DOX. Kinetics of EHT, proliferation, and apoptosis were analyzed from D4 + 1 to D4 + 6 of secondary culture.

(legend continued on next page)

Molecular Profiling of IG2$^{+/+}$ and IG2$^{-/-}$ Cells Revealed Unique Features of GATA2 Network that Distinguish Hemogenic Precursors from Non-HE

Our findings that GATA2 has little effect on the formation of HE and non-HE, and demonstrating that GATA2 has a selective EHT-inducing effect on HE, suggest that specification of HE and non-HE endothelial lineages is regulated by GATA2-independent pathways. These pathways likely predetermine the genetic and epigenetic landscape in which GATA2 may act. To support this hypothesis, we performed RNA-seq analysis of HE cultured with and without DOX, non-HE, and CD43^{+} cells from iG2$^{+/+}$ and iG2$^{-/-}$ hESCs. We found the most profound differences in gene expression in CD43^{+} cells. The total number of differentially expressed genes between iG2$^{+/+}$ and iG2$^{-/-}$ CD43^{+} blood was 1,701, while only 712 genes where differentially expressed in HE, and 761 in non-HE. Induction of GATA2 expression in iG2$^{-/-}$ and iG2$^{+/+}$ HE affected expression of approximately 1,400 genes (Figure S6A). Analysis of biological function of differentially expressed genes identified two main subcategories in the set of gene ontology (GO) cellular component categories: cell surface (including plasma membrane and extracellular matrix) and cytoskeleton. As shown in Figure 6A, iG2$^{+/+}$ CD43^{+} cells downregulated genes in categories associated with cell surface. In contrast, plasma membrane-associated categories were upregulated, while cytoskeleton-related categories were downregulated in IG2$^{+/+}$ HE. Enforced expression of GATA2 in iG2$^{-/-}$ and iG2$^{+/+}$ HE was also associated with gene downregulation in cell surface categories (Figure S6B). Differences in GO categories between iG2$^{-/-}$ and iG2$^{+/+}$ non-HE were less pronounced and included only a few GO categories associated with extracellular region. In addition, we found little overlap between genes differentially expressed in iG2$^{-/-}$ and iG2$^{+/+}$ HE and non-HE (only 16) (Figure S6A), thereby suggesting little commonality in molecular pathways affected by GATA2 in these two distinct endothelial populations. Using known transcription-target relationships, we constructed the *GATA2* gene regulatory network operating in HE, non-HE, and CD43^{+} cells. The relative abundance of mRNA expression in these networks was coded as node size, while color density represents enrichment (red) or depletion (blue) of known targets of that transcription factor (regulon members) among the differentially expressed genes. As shown in Figures 6B and S6C, this network revealed that upregulation of the *KLF1, NFE2*, and *GFI1B* genes and their regulons, was the most stable core of response across all hemogenic subsets. Besides having GATA2 as a common upstream regulator, they are heavily regulated by other factors (11–12 regulators per gene). Of those 11–12, eight (E2F2, GATA1, GATA2, GFI1B, LMO2, LYL1, MYB, and TAL1) are common upstream regulators for all three genes. Importantly, seven out of those eight known upstream regulators (LYL1 excluded) were selected as functionally relevant by our data-driven regulon selection procedure (see Supplemental Experimental Procedures). Another common feature of the GATA2 network in all hemogenic subsets was downregulation of the regulons for *SOX17, SOX18*, and *NOTCH1*. The observed changes in the described transcription factor activities within the GATA2 network were minimal at the HE stage, but became more pronounced at the CD43^{+} stage or following enforced expression of GATA2 in HE (Figures 6B and S6C). Assembly of the dynamic core regulatory network for hematopoietic specification based on multi-omics analysis of different stages of mouse ESC differentiation revealed an increase in transcription factor binding events at *GATA2* promoter following transition from HE to HP stage, thereby suggesting an increase in upstream regulation of GATA2 at the HP stage (Goode et al., 2016). Elevated level of GATA2 regulon activity following HE to HP transition in our studies indicates that increased upstream regulation of GATA2 during this transition is accompanied by upregulation of the GATA2 downstream network.

As shown in Figure 6B, the GATA2 transcriptional network in non-HE was very different. Compared with HE, *NFE2, KLF1*, and *GFI1B* regulons were not active, while, *SOX17* and *SOX18* regulons displayed slight increases in their regulon-level signal in non-HE. In addition, another distinctive feature of non-HE was upregulation of FOXM1, HMGA2, and HELLS mRNA and associated regulons, along with *TEAD3, TBX3, GATA6, NRF2*, and *GLIS2* regulons (Figure 6B). Thus, the observed differences between GATA2 transcriptional activities in HE and non-HE supports our hypothesis that GATA2 is most critical for enforcing hematopoietic program during EHT from HE, while mechanisms upstream of GATA2 are essential for specification of HE and non-HE from mesoderm, and for pre-establishing GATA2-responsive hematopoietic program in HE.

(D) Representative contour plots show EHT kinetics in the presence or absence of GATA2 during 6 days culture of D4 HE.
(E) Microscopic images display the failure of iG2$^{-/-}$ HE to undergo EHT. Scale bars, 100 μm.
(F and G) Percentages of CD43^{+} cells (F) and frequencies of hematopoietic CFCs (G) in D4 + 6 HE cultures with and without DOX.
(H) Analysis of the effect of GATA2 on blood production at single-cell level. Single D4 HE cells were FACS-sorted into 96-well plates with OP9 and cultured with or without DOX. Hematopoietic (HC), endothelial (EC), and mixed (MC) colonies were scored based on CD43 and VEC expression on D4 + 6 by immunofluorescence and counted by eye.
Scale bars, 100 μm. Bars in (B), (F), and (G) are means ± SE for at least three experiments. *$p<0.05$, **$p<0.01$. See also Figures S4 and S5.

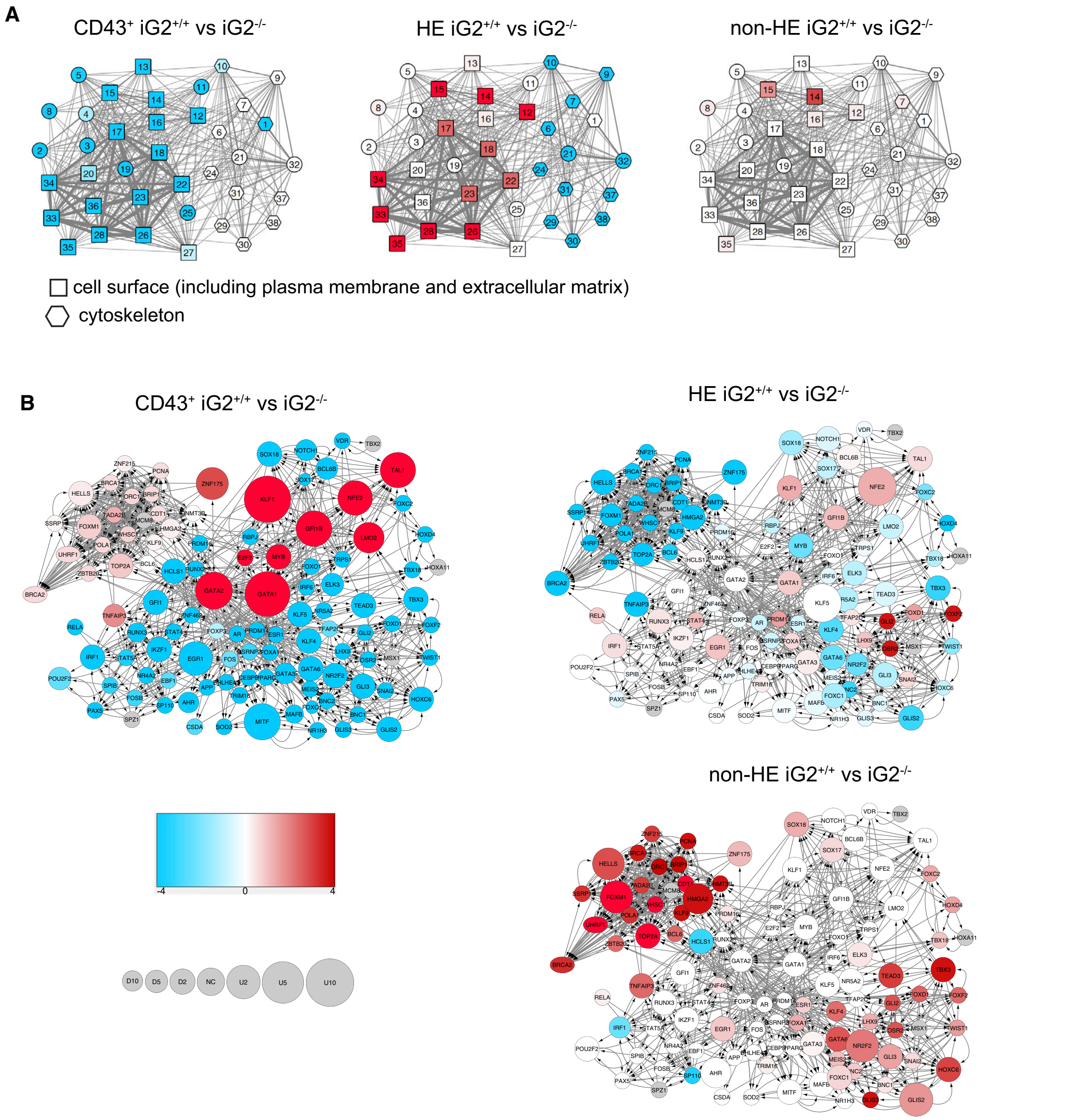

Figure 6. Gene Expression Profiling Reveals Distinct Features of GATA2 Regulatory Network during Hematopoietic Development

(A) Gene ontology analysis shows main gene ontology cellular component (GOCC) categories of cell surface (including plasma membrane and extracellular matrix) and cytoskeleton were found to be affected by GATA2 in the indicated cell subsets. Nodes representing those two supercategories are coded by shape, with squares representing cell surface and hexagons representing cytoskeleton. Node identification numbers correspond to the GOCC categories defined in Figure S6B. The color density represents enrichment (red) or depletion (blue) of differentially expressed genes related to displayed category. The width of the edges reflects the number of genes shared by categories.

(B) GATA2 transcriptional regulatory network reconstructed based on analysis of differentially expressed genes in $iG2^{+/+}$ and $iG2^{-/-}$ cells. Node size represents relative abundance of mRNA of the respective gene, computed as $\log_2$(fold change) in $iG2^{+/+}$ versus $iG2^{-/-}$ cells (see circle size scale below; U, upregulated; NC, no change; and D, downregulated). Both up- and downregulation effects are mapped onto the node size. The color density represents enrichment (red) or depletion (blue) of known targets of that transcription factor (regulon

(legend continued on next page)

DISCUSSION

In the present study, we used *GATA2* knockout hESC lines, with conditional GATA2 expression, to define the exact role of GATA2 at the HE stage of hematopoietic development. Although blood formation from HE through EHT is well documented, the exact sequence of events and the molecular mechanisms leading to blood specification from mesoderm is not well understood. Avian studies have demonstrated that HE and non-HE in the aorta arises from different mesodermal populations (Pardanaud and Dieterlen-Lievre, 1999; Pardanaud et al., 1996; Pouget et al., 2006). In addition, demonstration that HE population from mouse AGM produces only endothelial or blood cells (Swiers et al., 2013a) suggests that HE represents a unique blood-forming endothelial lineage, which is likely specified independently of non-HE. This hypothesis is also supported by our findings that at least two distinct types of endothelial mesodermal progenitors with mesenchymal and hematopoietic potentials are established in hPSC cultures, and that endothelial cells with hemogenic potential possess a distinct $CD73^{-}$ phenotype (Choi et al., 2012; Vodyanik et al., 2010). Thus, perturbation of blood development at hemogenic sites can be caused by the selective effect of transcription factors on HE specification, EHT per se, or alternatively by their effect on amplification, survival, and specification of blood progenitors at the post-EHT stage. Among transcription factors involved in hematopoietic development, Runx1 has been shown to specify endothelial cells as hemogenic during a very short developmental window (Yzaguirre et al., 2018). In addition, Runx1 affects post-EHT stages of blood development, including transition of $VEC^{+}CD45^{-}CD41^{+}$ type I HSCs to the $CD45^{+}$ type II HSCs (Liakhovitskaia et al., 2014). Double knockout of Gfi1 and Gfi1b proteins abrogates formation of intraortic hematopoietic clusters, but HE cells can still be detected in the ventral domain of dorsal aorta (Thambyrajah et al., 2016a). In the embryo, GATA2 expression is initiated in the primitive streak. Later, GATA2 expression is found in lateral plate mesoderm and at sites of embryonic hematopoiesis including the yolk sac, endothelial lining of dorsal aorta, vitelline and umbilical arteries, intra-aortic hematopoietic clusters and in fetal liver HSCs (Minegishi et al., 1999, 2003). *Xenopus* studies suggest that GATA2 may act in a cell-autonomous manner to promote hematopoietic specification at the mesodermal stage, while functioning in ectodermal and stromal layers in a cell-non-autonomous manner to promote hematopoietic differentiation (Maeno et al., 1996). In mice, conditional knockout of *GATA2* in VEC^{+} cells and analysis of AGM hematopoiesis in mice have demonstrated the essential role of GATA2 during EHT and post-EHT (de Pater et al., 2013; Gao et al., 2013; Lim et al., 2012). Studies by Mikolla's group using *Gata1* and *Gata2* double knockout mouse ESCs revealed that these cells, in contrast to *Scl* knockout ESCs, can differentiate into $Flk1^{+}Tie2^{+}CD31^{+}CD41^{+}c\text{-}kit^{-}$ cells, raising the possibility that the absence of *Gata1* and *Gata2* does not prevent HE formation (Org et al., 2015). However, whether GATA2 solely affects EHT without having any effect on mesoderm or HE diversification remains unclear. Herein, using engineered *GATA2* knockout hESCs with conditional GATA2 expression, we demonstrated that GATA2 has little effect on specification of mesodermal and endothelial lineages at pre-hematopoietic fate. The development of $APLNR^{+}PDGFR\alpha^{+}$ primitive posterior mesodermal cells, and the more committed $KDR^{hi}VEC^{-}$ hematovascular mesodermal progenitors, were not affected by GATA2, and these cells retained endothelial potential in $iG2^{-/-}$ cultures. Formation of non-HE and HE, and subsequent HE specification to $DLL4^{+}$ arterial and $DLL4^{-}$ non-arterial HE, was not affected by GATA2 either. However, critical factors involved in EHT, GFI1, and RUNX1 (Chen et al., 2009; Thambyrajah et al., 2016a) were downregulated in $iG2^{-/-}$ HE compared with $iG2^{+/+}$ HE, and $iG2^{-/-}$ HE failed to undergo EHT. Following restoration of GATA2 expression in $iG2^{-/-}$ HE cells, they regained the ability to undergo EHT and blood formation. Thus, we have provided direct evidence that GATA2 endows hematopoietic activity predominantly through promotion of EHT, but not HE formation.

GATA2 hemogenic activity was very specific for HE. Forced expression of GATA2 in non-HE failed to induce significant blood production, which suggests that the hematopoietic program on which GATA2 may act is likely pre-established by other factors during HE specification. This conclusion is also supported by our demonstration of cell-specific differences in the GATA2 network within HE and non-HE.

We also revealed that *GATA2* knockout hESCs are still able to produce a small number of $CD43^{+}$ HPs. These HPs have markedly diminished granulocytic and erythroid potentials, but are still capable of macrophage, T, and NK cell differentiation. Accumulating evidence suggests that hematopoiesis in the absence of GATA2 can be supported

members) among the differentially expressed genes. Color scale: numbers are signed log-transformed false discovery rate (FDR) values $-\log_{10}(FDR)$ for upregulation (positive numbers), $\log_{10}(FDR)$ for downregulation (negative numbers). Network visualization was performed using Cytoscape v.3.4.0.
See also Figure S6.

through action of other GATA factors. Studies by the Munoz-Chapuli group revealed a subset of HSCs arising from progenitors expressing Gata4 under control of G2 mesodermal-specific *Gata4* enhancer located in placenta and lateral plate mesoderm (Canete et al., 2017). In addition, molecular profiling studies of Gata2-negative HPs demonstrated upregulation of Gata3 and Gata4, suggesting that these Gata factors may provide some function in Gata2-independent hematopoietic cells factors (Kaimakis et al., 2016). Analysis of various GATA factors in our studies have revealed that $iG2^{-/-}$ $CD43^{+}$ cells express higher levels of *GATA3, GATA4, GATA5*, and *GATA6,* thereby suggesting that they may resemble GATA2-independent HPs recently described in mouse.

In summary, our study provides a new dimension into how GATA2 promotes blood development during EHT. The lack of a GATA2 requirement for HE generation raises important questions regarding the nature of the molecular mechanisms that function upstream of GATA2. Among these mechanisms, ETV2-mediated hematopoietic programming could be the most significant. Transient expression of ETV2 at the mesodermal stage establishes a lineage-specific epigenetic landscape in the blood and vascular system; activates a network of hematoendothelial transcription factors; and, together with vascular endothelial growth factor A (VEGFA) and FLK1, forms a key regulatory module in hemangiogenic fate commitment (Kataoka et al., 2011; Liu et al., 2015; Wareing et al., 2012; Zhao and Choi, 2017). ETV2 interacts with GATA2 and upregulates GATA2 expression in undifferentiated hESCs (Elcheva et al., 2014; Shi et al., 2014). In addition, the coexpression of both factors can directly induce HE development from hPSCs and blood formation through EHT (Elcheva et al., 2014). Exploring the precise molecular mechanisms guiding HE and blood specification upstream and downstream of GATA2 will help to facilitate new technologies for scalable blood cell production from hPSCs or through direct cellular reprogramming of somatic cells for use in transfusion and immunotherapies.

EXPERIMENTAL PROCEDURES

Cell Culture

WA01 (H1) hESCs and genetically modified hESCs generated in this study (see Supplemental Experimental Procedures) were maintained on Matrigel-coated plates in E8 medium (Chen et al., 2011). Cells were passaged using 0.5 mM EDTA in PBS when they reached to around 85%–90% (4–5 days) confluency.

Hematopoietic Differentiation of hESCs

Hematopoietic differentiation was performed on type IV collagen (ColIV) (Sigma-Aldrich)-coated plates in E8 medium according to a previously described protocol (Uenishi et al., 2014). To induce exogenous GATA2 expression, 5 μg/mL DOX was added at the day of interest for 24 hr. Differentiation efficiency was assessed at day 8 of differentiation by flow cytometry and CFC assay.

Isolation and Culture of HE

$VEC^{+}CD43^{-}CD73^{-}$ HE was isolated on day 4 of differentiation using $CD31^{+}$ antibodies and magnetic-activated cell sorting or fluorescence-activated cell sorting (FACS) (see Supplemental Experimental Procedures for details). HE cells were plated on ColIV-coated 6- or 12-well plates at a density ranging from 20,000 to 30,000 cells/cm^2 in IF9S medium supplemented with 50 ng/mL FGF-2, 50 ng/mL VEGF, 50 ng/mL stem cell factor (SCF), 50 ng/mL interleukin-6 (IL-6), 50 ng/mL thyroperoxidase (TPO), 10 ng/mL IL-3, 50 ng/mL insulin growth factor 1 (IGF-1), 50 ng/mL IGF-2, 50 ng/mL epidermal growth factor, and 10 μM ROCKi (Uenishi et al., 2014). Where indicated, DOX at a concentration of 5 μg/mL was added during the first 1 or 2 days of secondary culture. Hematopoietic differentiation was evaluated by flow cytometry and CFC assay on day 6 of differentiation (day 4 + 6). In addition, at days 2, 4, and 6 of the secondary differentiation (day 4 + 2, 4, 6), cells were harvested and stained with anti-Ki67 antibody and annexin V/7AAD to assess proliferation and cell death.

Single-Cell Deposition Assay for EHT

Day 4 differentiated hPSCs were singularized, stained for CD31, and single-cell sorted into individual wells of the 96-well plates containing OP9 feeders using a FACS Aria II. HE cells were cultured for up to 6 days in alpha-MEM supplemented with 10% FBS, 50 ng/mL SCF, 50 ng/mL TPO, 10 ng/mL IL-3, and 20 ng/mL of IL-6 with/without DOX. DOX was removed after 2 days of culture. Fresh medium with cytokines was provided every other day. Culture plates were fixed and stained with anti-CD144 (rabbit, eBioscience) and anti-CD43 (mouse, BD Biosciences) primary antibodies, and anti-rabbit Alexa Fluor 488 and anti-mouse Alexa Fluor 594 secondary antibodies (Jackson Immunology). Hematopoietic, endothelial, and hematoendothelial clusters were observed under fluorescent microscope, and the ratio of hematopoietic clusters relative to endothelial clusters was calculated.

Statistical Analyses

Statistical analysis was performed using MASS package running in R programming language v.3.4.3. F homogeneity of variance test (F test) was performed first, and, depending on the result of the F test, either Student's t test or Welch's t test were conducted for statistical significance. Data were expressed as the mean ± SEM.

ACCESSION NUMBERS

The accession number for the RNA-seq data reported in this paper is GEO: GSE102767.

SUPPLEMENTAL INFORMATION

Supplemental Information includes Supplemental Experimental Procedures, six figures, three tables, and one data file and can be found with this article online at https://doi.org/10.1016/j.stemcr.2018.05.002.

AUTHOR CONTRIBUTIONS

H.J.K. designed, conducted, and analyzed the experiments, interpreted the experimental data, made the figures, and contributed to the concept and manuscript writing. W.-T.M. performed NK cell differentiation studies. O.V.M. performed RNA-seq bioinformatics analysis. H.S.J. preformed cell-cycle analysis. J.A.T. directed the RNA-seq studies. I.I.S. developed the concept, led and supervised the studies, analyzed and interpreted the data, and wrote the manuscript.

ACKNOWLEDGMENTS

We thank Dr. Emery Bresnick (University of Wisconsin) for providing GATA2 antibodies and for comments on the manuscript, Koichi R Katsumura (Bresnick lab) for troubleshooting GATA2 detection by western blot, and Matthew Raymond for editorial assistance. This work was supported by funds from the NIH (R01HL116221, U01HL099773, and P51 RR000167) and The Charlotte Geyer Foundation.

Received: December 15, 2017
Revised: May 2, 2018
Accepted: May 3, 2018
Published: May 31, 2018

REFERENCES

Canete, A., Carmona, R., Ariza, L., Sanchez, M.J., Rojas, A., and Munoz-Chapuli, R. (2017). A population of hematopoietic stem cells derives from GATA4-expressing progenitors located in the placenta and lateral mesoderm of mice. Haematologica *102*, 647–655.

Chen, M.J., Yokomizo, T., Zeigler, B.M., Dzierzak, E., and Speck, N.A. (2009). Runx1 is required for the endothelial to haematopoietic cell transition but not thereafter. Nature *457*, 887–891.

Chen, G., Gulbranson, D.R., Hou, Z., Bolin, J.M., Ruotti, V., Probasco, M.D., Smuga-Otto, K., Howden, S.E., Diol, N.R., Propson, N.E., et al. (2011). Chemically defined conditions for human iPSC derivation and culture. Nat. Methods *8*, 424–429.

Choi, K.D., Vodyanik, M.A., and Slukvin, I.I. (2009a). Generation of mature human myelomonocytic cells through expansion and differentiation of pluripotent stem cell-derived lin-CD34+CD43+CD45+ progenitors. J. Clin. Invest. *119*, 2818–2829.

Choi, K.D., Yu, J., Smuga-Otto, K., Salvagiotto, G., Rehrauer, W., Vodyanik, M., Thomson, J., and Slukvin, I. (2009b). Hematopoietic and endothelial differentiation of human induced pluripotent stem cells. Stem Cells *27*, 559–567.

Choi, K.D., Vodyanik, M.A., Togarrati, P.P., Suknuntha, K., Kumar, A., Samarjeet, F., Probasco, M.D., Tian, S., Stewart, R., Thomson, J.A., et al. (2012). Identification of the hemogenic endothelial progenitor and its direct precursor in human pluripotent stem cell differentiation cultures. Cell Rep. *2*, 553–567.

de Pater, E., Kaimakis, P., Vink, C.S., Yokomizo, T., Yamada-Inagawa, T., van der Linden, R., Kartalaei, P.S., Camper, S.A., Speck, N., and Dzierzak, E. (2013). Gata2 is required for HSC generation and survival. J. Exp. Med. *210*, 2843–2850.

Ditadi, A., Sturgeon, C.M., Tober, J., Awong, G., Kennedy, M., Yzaguirre, A.D., Azzola, L., Ng, E.S., Stanley, E.G., French, D.L., et al. (2015). Human definitive haemogenic endothelium and arterial vascular endothelium represent distinct lineages. Nat. Cell Biol. *17*, 580–591.

Eich, C., Arlt, J., Vink, C.S., Solaimani Kartalaei, P., Kaimakis, P., Mariani, S.A., van der Linden, R., van Cappellen, W.A., and Dzierzak, E. (2018). In vivo single cell analysis reveals Gata2 dynamics in cells transitioning to hematopoietic fate. J. Exp. Med. *215*, 233–248.

Elcheva, I., Brok-Volchanskaya, V., Kumar, A., Liu, P., Lee, J.H., Tong, L., Vodyanik, M., Swanson, S., Stewart, R., Kyba, M., et al. (2014). Direct induction of haematoendothelial programs in human pluripotent stem cells by transcriptional regulators. Nat. Commun. *5*, 4372.

Gao, X., Johnson, K.D., Chang, Y.I., Boyer, M.E., Dewey, C.N., Zhang, J., and Bresnick, E.H. (2013). Gata2 cis-element is required for hematopoietic stem cell generation in the mammalian embryo. J. Exp. Med. *210*, 2833–2842.

Goode, D.K., Obier, N., Vijayabaskar, M.S., Lie, A.L.M., Lilly, A.J., Hannah, R., Lichtinger, M., Batta, K., Florkowska, M., Patel, R., et al. (2016). Dynamic gene regulatory networks drive hematopoietic specification and differentiation. Dev. Cell *36*, 572–587.

Guibentif, C., Ronn, R.E., Boiers, C., Lang, S., Saxena, S., Soneji, S., Enver, T., Karlsson, G., and Woods, N.B. (2017). Single-cell analysis identifies distinct stages of human endothelial-to-hematopoietic transition. Cell Rep. *19*, 10–19.

Huang, K., Du, J., Ma, N., Liu, J., Wu, P., Dong, X., Meng, M., Wang, W., Chen, X., Shi, X., et al. (2015). GATA2(–/–) human ESCs undergo attenuated endothelial to hematopoietic transition and thereafter granulocyte commitment. Cell Regen. (Lond.) *4*, 4.

Jaffredo, T., Richard, C., Pouget, C., Teillet, M.A., Bollerot, K., Gautier, R., and Drevon, C. (2010). Aortic remodelling during hemogenesis: is the chicken paradigm unique? Int. J. Dev. Biol. *54*, 1045–1054.

Kaimakis, P., de Pater, E., Eich, C., Solaimani Kartalaei, P., Kauts, M.L., Vink, C.S., van der Linden, R., Jaegle, M., Yokomizo, T., Meijer, D., et al. (2016). Functional and molecular characterization of mouse Gata2-independent hematopoietic progenitors. Blood *127*, 1426–1437.

Kataoka, H., Hayashi, M., Nakagawa, R., Tanaka, Y., Izumi, N., Nishikawa, S., Jakt, M.L., Tarui, H., and Nishikawa, S. (2011). Etv2/ER71 induces vascular mesoderm from Flk1+PDGFRalpha+ primitive mesoderm. Blood *118*, 6975–6986.

Lancrin, C., Sroczynska, P., Stephenson, C., Allen, T., Kouskoff, V., and Lacaud, G. (2009). The haemangioblast generates haematopoietic cells through a haemogenic endothelium stage. Nature *457*, 892–895.

Liakhovitskaia, A., Rybtsov, S., Smith, T., Batsivari, A., Rybtsova, N., Rode, C., de Bruijn, M., Buchholz, F., Gordon-Keylock, S., Zhao, S., et al. (2014). Runx1 is required for progression of CD41+ embryonic precursors into HSCs but not prior to this. Development *141*, 3319–3323.

Lim, K.C., Hosoya, T., Brandt, W., Ku, C.J., Hosoya-Ohmura, S., Camper, S.A., Yamamoto, M., and Engel, J.D. (2012). Conditional

Gata2 inactivation results in HSC loss and lymphatic mispatterning. J. Clin. Invest. *122*, 3705–3717.

Liu, F., Li, D., Yu, Y.Y., Kang, I., Cha, M.J., Kim, J.Y., Park, C., Watson, D.K., Wang, T., and Choi, K. (2015). Induction of hematopoietic and endothelial cell program orchestrated by ETS transcription factor ER71/ETV2. EMBO Rep. *16*, 654–669.

Maeno, M., Mead, P.E., Kelley, C., Xu, R.H., Kung, H.F., Suzuki, A., Ueno, N., and Zon, L.I. (1996). The role of BMP-4 and GATA-2 in the induction and differentiation of hematopoietic mesoderm in *Xenopus laevis*. Blood *88*, 1965–1972.

Minegishi, N., Ohta, J., Yamagiwa, H., Suzuki, N., Kawauchi, S., Zhou, Y.H., Takahashi, S., Hayashi, N., Engel, J.D., and Yamamoto, M. (1999). The mouse GATA-2 gene is expressed in the para-aortic splanchnopleura and aorta-gonads and mesonephros region. Blood *93*, 4196–4207.

Minegishi, N., Suzuki, N., Yokomizo, T., Pan, X., Fujimoto, T., Takahashi, S., Hara, T., Miyajima, A., Nishikawa, S., and Yamamoto, M. (2003). Expression and domain-specific function of GATA-2 during differentiation of the hematopoietic precursor cells in midgestation mouse embryos. Blood *102*, 896–905.

North, T., Gu, T.L., Stacy, T., Wang, Q., Howard, L., Binder, M., Marin-Padilla, M., and Speck, N.A. (1999). Cbfa2 is required for the formation of intra-aortic hematopoietic clusters. Development *126*, 2563–2575.

Org, T., Duan, D., Ferrari, R., Montel-Hagen, A., Van Handel, B., Kerenyi, M.A., Sasidharan, R., Rubbi, L., Fujiwara, Y., Pellegrini, M., et al. (2015). Scl binds to primed enhancers in mesoderm to regulate hematopoietic and cardiac fate divergence. EMBO J. *34*, 759–777.

Pardanaud, L., and Dieterlen-Lievre, F. (1999). Manipulation of the angiopoietic/hemangiopoietic commitment in the avian embryo. Development *126*, 617–627.

Pardanaud, L., Luton, D., Prigent, M., Bourcheix, L.M., Catala, M., and Dieterlen-Lievre, F. (1996). Two distinct endothelial lineages in ontogeny, one of them related to hemopoiesis. Development *122*, 1363–1371.

Pouget, C., Gautier, R., Teillet, M.A., and Jaffredo, T. (2006). Somite-derived cells replace ventral aortic hemangioblasts and provide aortic smooth muscle cells of the trunk. Development *133*, 1013–1022.

Shi, X., Richard, J., Zirbes, K.M., Gong, W., Lin, G., Kyba, M., Thomson, J.A., Koyano-Nakagawa, N., and Garry, D.J. (2014). Cooperative interaction of Etv2 and Gata2 regulates the development of endothelial and hematopoietic lineages. Dev. Biol. *389*, 208–218.

Slukvin, I.I. (2016). Generating human hematopoietic stem cells in vitro - exploring endothelial to hematopoietic transition as a portal for stemness acquisition. FEBS Lett. *590*, 4126–4143.

Swiers, G., Baumann, C., O'Rourke, J., Giannoulatou, E., Taylor, S., Joshi, A., Moignard, V., Pina, C., Bee, T., Kokkaliaris, K.D., et al. (2013a). Early dynamic fate changes in haemogenic endothelium characterized at the single-cell level. Nat. Commun. *4*, 2924.

Swiers, G., Rode, C., Azzoni, E., and de Bruijn, M.F. (2013b). A short history of hemogenic endothelium. Blood Cells Mol. Dis. *51*, 206–212.

Thambyrajah, R., Mazan, M., Patel, R., Moignard, V., Stefanska, M., Marinopoulou, E., Li, Y., Lancrin, C., Clapes, T., Moroy, T., et al. (2016a). GFI1 proteins orchestrate the emergence of haematopoietic stem cells through recruitment of LSD1. Nat. Cell Biol. *18*, 21–32.

Thambyrajah, R., Patel, R., Mazan, M., Lie, A.L.M., Lilly, A., Eliades, A., Menegatti, S., Garcia-Alegria, E., Florkowska, M., Batta, K., et al. (2016b). New insights into the regulation by RUNX1 and GFI1(s) proteins of the endothelial to hematopoietic transition generating primordial hematopoietic cells. Cell Cycle *15*, 2108–2114.

Tsai, F.Y., Keller, G., Kuo, F.C., Weiss, M., Chen, J., Rosenblatt, M., Alt, F.W., and Orkin, S.H. (1994). An early haematopoietic defect in mice lacking the transcription factor GATA-2. Nature *371*, 221–226.

Tsai, F.Y., and Orkin, S.H. (1997). Transcription factor GATA-2 is required for proliferation/survival of early hematopoietic cells and mast cell formation, but not for erythroid and myeloid terminal differentiation. Blood *89*, 3636–3643.

Uenishi, G., Theisen, D., Lee, J.H., Kumar, A., Raymond, M., Vodyanik, M., Swanson, S., Stewart, R., Thomson, J., and Slukvin, I. (2014). Tenascin C promotes hematoendothelial development and T lymphoid commitment from human pluripotent stem cells in chemically defined conditions. Stem Cell Rep. *3*, 1073–1084.

Uenishi, G.I., Jung, H.S., Kumar, A., Park, M.A., Hadland, B., McLeod, E., Raymond, M., Moskvin, O.V., Zimmerman, C., Theisen, D.J., et al. (2018). NOTCH signaling specifies arterial-type definitive hemogenic endothelium from human pluripotent stem cells. Nat. Commun. *9*, 1828.

Vodyanik, M.A., Thomson, J.A., and Slukvin, I.I. (2006). Leukosialin (CD43) defines hematopoietic progenitors in human embryonic stem cell differentiation cultures. Blood *108*, 2095–2105.

Vodyanik, M.A., Yu, J., Zhang, X., Tian, S., Stewart, R., Thomson, J.A., and Slukvin, I.I. (2010). A mesoderm-derived precursor for mesenchymal stem and endothelial cells. Cell Stem Cell *7*, 718–729.

Wareing, S., Mazan, A., Pearson, S., Gottgens, B., Lacaud, G., and Kouskoff, V. (2012). The Flk1-Cre-mediated deletion of ETV2 defines its narrow temporal requirement during embryonic hematopoietic development. Stem Cells *30*, 1521–1531.

Yzaguirre, A.D., Howell, E.D., Li, Y., Liu, Z., and Speck, N.A. (2018). Runx1 is sufficient for blood cell formation from non-hemogenic endothelial cells in vivo only during early embryogenesis. Development *145*. https://doi.org/10.1242/dev.158162.

Zhao, H., and Choi, K. (2017). A CRISPR screen identifies genes controlling Etv2 threshold expression in murine hemangiogenic fate commitment. Nat. Commun. *8*, 541.

Stem Cell Reports, Volume *11*

Supplemental Information

GATA2 Is Dispensable for Specification of Hemogenic Endothelium but Promotes Endothelial-to-Hematopoietic Transition

HyunJun Kang, Walatta-Tseyon Mesquitta, Ho Sun Jung, Oleg V. Moskvin, James A. Thomson, and Igor I. Slukvin

Supplementary Information
Kang et al., GATA2 is Dispensable for Specification of Hemogenic Endothelium but Required for Endothelial-to-Hematopoietic Transition

Supplemental Experimental Procedures

Construction of inducible hGATA2-expression vector
TET response element (TRE) containing sequence of seven tetracycline operators were connected with mouse beta globin minimal promoter (mBGMP). mBGMP-TRE was inserted into PiggyBac (PB) plasmid DNA, generating a doxycycline-inducible PB-ipKTRE construct. CDS (coding domain sequence) of the human *GATA2* gene with a HA-tag in the 5'-region was connected with P2A followed by EGFP as a reporter, generating HA-GATA2-P2A-EGFP fragment, which expresses *GATA2* and *EGFP* in a bicistronic manner. Then, the HA-hGATA2-P2A-EGFP fragment was inserted into the mBGMP-TRE construct, thus generating the PB- mBGMP-TRE-HA-hGATA2-P2A-EGFP (PB-iGATA2 hereafter) construct (Figure S1). As one of the trans-activating components for TRE, an optimized form of the CDS of reverse tetracycline-controlled transactivator (*M2rtTA*) was put under a EF1alpha promoter followed by T2A and puromycin resistance (*PuroR*) gene as a selection marker, generating EF1alpha-M2rtTA-T2A-PuroR fragment, which was then inserted into PB plasmid DNA (PB-EF1alpha-M2rtTA-T2A-PuroR or PB-M2rtTA) construct (Figure S1).

Generation of intact GATA2-inducible H1 hESC (iG2$^{+/+}$) line
H1 cells growing on Matrigel in E8 media were treated with 10 μM ROCK inhibitor (ROCKi; Tocris Y-27632) 1 hour prior to nucleofection and detached by TrypLE Select (LifeTech) and singularized by pipetting. Approximately 5×10^5 cells of the single cell suspension were resuspended in 100 μL of Human Stem Cell Nucleofector Solution 1 (Lonza) containing 18 uL of Supplement 1 solution (Lonza), 5 μg of PB-iGATA2 construct, 5 μg of PB-M2rtTA construct and 1 μg of transposase-expressing plasmid DNA. Nucleofection was carried out according to manufacturer's instruction (Lonza). After transfection, cells were replated on Matrigel with E8 media containing 10 μM ROCKi and placed in 37 °C with 5% CO_2. Fresh E8 media without ROCKi was provided daily. Puromycin was added to the media at a concentration of 0.5 - 1 μg/mL around 3 - 4 days after nucleofection. Puromycin-resistant colonies were picked and expanded for further analysis and downstream experiments. Induction of transgene (GATA2 and EGFP) expression was tested by adding DOX into culture media and detecting EGFP expression by fluorescent microscopy and flow cytometry. Finally, selected colonies are referred to as intact endogenous GATA2-inducible H1 hESC (iG2$^{+/+}$) cells.

Construction of GATA2-targeting vectors
According to the general rules (Mali et al., 2013), four crRNA sequences were designed to target introns near both ends of exon 2 coding signal peptides and transactivation domain (crRNA1 & 2), and exon 5 coding zinc finger motifs for DNA

binding (crRNA3 & 4) with the goal to excise both exons (Figure 1B). These crRNAs were PCR-amplified and inserted downstream of U6 polymerase III promoter and upstream tracrRNA sequence in gRNA expression vector, MLM3636 (Addgene plasmids 43860 from Keith Joung), generating gRNA1-4 from crRNA1-4, respectively. For Cas9 protein, mammalian codon-optimized Cas9-coding plasmid DNA, JDS246 (Addgene plasmids 43861 from Keith Joung), was used.

Generation of endogenous GATA2 knockout exogenous GATA2-inducible (iG2$^{-/-}$) H1 hESC line

One hour prior to nucleofection, the iG2$^{+/+}$ cells growing on Matrigel in E8 were treated with 10 μM ROCKi, detached by TrypLE Select (LifeTech), and singularized by pipetting. 1×10^6 cells were then resuspended in 100 μL of Human Stem Cell Nucleofector Solution 1 containing 18 μL of Supplement 1 solution and 1.5 μg each of the gRNAs (gRNA1-4) and 20 μg of JDS246, and then transfected according to the manufacturer instruction (Lonza). Transfected cells were cultured on Matrigel-coated plate with E8 containing 10 μM ROCKi at 37 °C with 5% CO2. Fresh E8 media without ROCKi was provided every day. Four to five days later, single colonies were picked up and expanded. To identify and confirm a biallelic mutation in *GATA2* gene, genomic DNAs were extracted from expanded individual clones and PCR-amplified with primers specifically binding to targeting areas (Table S2). Selected mutant clones were subjected to single cell sorting using FACSAria II (BD Biosciences) to obtain single cell-derived colonies, thus ensuring the homogeneity of mutant cell lines. Genomic DNAs were extracted and PCR-amplified from single cell-derived lines to amplify and clone PCR products into a T vector for sequencing. From the sequencing results, two endogeneous GATA2 knockout exogenous GATA2-inducible cell lines, iG2$^{-/-}$SC3 and iG2$^{-/-}$SC6, were obtained and used for further experiments.

Confirming pluripotency of genetically modified hESC lines

To confirm pluripotency, flow cytometry analysis with anti-SSEA4 (Stemgent, Cambridge, MA), anti-TRA-1-60, anti-TRA-1-81 (Stemgent, MA), and anti-OCT4, anti-NANOG, and anti-SOX2 antibodies (BD Biosciences, NJ) against pluripotent markers was performed. For teratoma formation, hESCs were harvested in 100 μL of 30% Matrigel in DMEM/F12 basal media, and then injected subcutaneously into the hind leg of NOD.Cg-Prkdcscidll2rgtm1wjl/SzJ mice (The Jackson Laboratory, ME). Around 8 – 12 weeks post-injection, teratomas were collected for microscopic examination.

MACS sorting

Cells of interest that were harvested from whole differentiation cultures were subjected to MACS with the appropriated flourochrome-conjugated antibodies and corresponding magnetic microbeads. The purity of isolated fractions as verified by flow cytometry was more than 95%.

Hemangioblast and hematopoietic CFC assays

Hemangioblast assay was performed in a serum-free semisolid medium supplemented with FGF2 as previously described (Vodyanik et al., 2010). Hematopoietic CFCs were detected using H4436 MethoCult (Stem Cell Technologies) according to the manufacturer.

T cell differentiation
$CD43^+$ hematopoietic progenitors were sorted from day 8 or 9 of differentiation by MACS, and then plated on a monolayer of OP9 expressing dim/modest level of human DLL4 ($OP9^{dim}$) (Kaimakis et al., 2016) in initial T cell differentiation media (IniTDM) consisting of alpha-MEM (GIBCO) supplemented with 20% FBS (Hyclone), 50 μg/mL of ascorbic acid, 50 ng/mL FLT3L, 50 ng/mL SCF, and 20 ng/mL IL-7. The second week, T cell differentiation media was composed of alpha-MEM supplemented with 20% FBS, 50 μg/mL ascorbic acid, 20 ng/mL FLT3L, 20 ng/mL SCF, 5 ng/mL IL-7; and the third week T cell differentiation media consisted of alpha-MEM supplemented with 20% FBS, 50 μg/mL ascorbic acid, 10 ng/mL FLT3L, 20 ng/mL SCF, 5 ng/mL IL-7, 25 ng/mL IL-2. Differentiating cells were harvested using 1 mg/mL of collagenase IV (GIBCO) solution and transferred to a fresh monolayer of OP9-DLL4 weekly with the appropriate T cell differentiation media added once per week. At days 21 - 25, whole cells were harvested and analyzed by flow cytometry.

NK cell differentiation
$CD43^+$ cells from day 8 or 9 of differentiation cultures were isolated by MACS and plated on a monolayer of $OP9\text{-}DLL4^{dim}$ cells in NK cell differentiation media (NKDM) consisting of alpha-MEM supplemented with 20% Hyclone FBS, 10 ng/mL FLT3L, 40 ng/mL SCF, and 25 ng/mL IL-7. One week later, cells were transferred onto a fresh monolayer of $OP9\text{-}DLL4^{dim}$ cells in NKDM with 10 ng/mL IL-2 and 5 ng/mL IL-15. Fresh NKDM containing IL-2 and IL-15, but without IL-3, was provided every three days. Cells were harvested at days 21-25 and analyzed by flow cytometry with CD94 and CD56 after gating $CD45^+$ cells.

Flow cytometry and FACS
Flow cytometry was performed using MACSQuant® Analyzer 10 (Miltenyi Biotec) and the following antibodies: CD4-APC (RPA-T4), CD31-FITC (WM59), CD41a-APC (HIP8), CD43-APC (1G10), CD45-PE (5B1), CD73-PE (AD2), CD94-FITC (HP-3D9), CD235a-APC (GA-R2), KDR-PE (89106), and PDGFRa-PE (aR1) from BD Biosciences, CD8-Vioblue (BW135), CD43-APC-Vio770 (DF-T1), CD45-PE-Vio770 (5B1), CD144-Vioblue (REA199), and DLL4-PE-Vio770 (MHD4-46) all from Miltenyi Biotec, CD56-PerCP (HCD56) from BioLegend, and APLNR-APC (72133) from R&D Systems. Appropriate isotype-matched mouse monoclonal antibodies as flourochrome controls were included in order to establish a threshold for the positive cell population and subsequent subset gating. FACS Aria (BD) was used for cell sorting.

Western blot

Proteins were isolated using Pierce IP Lysis Buffer (Thermo Fisher Scientific) supplemented with protease inhibitor cocktail (Sigma) from day 5 of hematopoietic differentiation. Isolated proteins were boiled for 5 minutes before loading on SDS-PAGE gel. GATA2 was detected with anti-GATA2 polyclonal antibody (Grass et al., 2006) kindly provided by Dr. Emery H. Bresnick (University of Wisconsin). Anti-GAPDH antibody (Santa Cruz Biotechnology) served as loading control. Proteins on blotting membrane were detected by ECL detection reagent (GE Healthcare).

RNA isolation

RNA was isolated using RNeasy Mini Kit (Qiagen) from cell populations of interest at different time points during differentiation, and DNA digestion was performed using RNase-Free DNase Set (Qiagen) during RNA isolation according to the manufacturer's instruction protocol. Isolated RNAs were used for either quantitative RT-PCR or RNAseq. Details about qPCR, RNAseq and bioinformatics analysis can be found in Supplemental Methods.

Quantitative RT-PCR

First strand cDNAs were synthesized using MMLV reverse transcriptase (ClonTech) and then 2 μL of the first strand cDNAs were subject to RT-PCR with SYBR Advantage qPCR Premix (ClonTech). RNA levels were normalized to RPL13A or GAPDH levels, and then relative levels between samples of interest were calculated. Primer sequences are provided in Table S3.

Low level RNA-Seq data processing

Total RNA was isolated from the day 4 $iG2^{+/+}$ and $iG2^{-/-}$ HE, day 4+1 $iG2^{+/+}$ and $iG2^{-/-}$ HE cultured with and without DOX, day 6 $iG2^{+/+}$ and $iG2^{-/-}$ $VEC^{+}CD43^{-}CD73^{+}$ nonHE and day 8 $iG2^{+/+}$ and $iG2^{-/-}$ $CD43^{+}$ cells. RNA purity and integrity was evaluated by capillary electrophoresis on the Bioanalyzer 2100 (Agilent Technologies, Santa Clara, CA). Samples were then prepared for sequencing using the Ligation Mediated Sequencing (LM-Seq) protocol, according to the published guidelines (Hou et al., 2015). Final sample libraries were quantitated with the Life Technologies Qubit fluorometer and sequenced on the Illumina HiSeq 3000 (SY-401-3001). Base-calling and demultiplexing were completed with the Illumina bcl2fastq2 utility, v2.17.1.14. Following quality assessment and filtering for adapter molecules and other sequencing artifacts, the sequencing reads were aligned to transcript sequences corresponding to hg19 human genome annotation. Bowtie v 1.1.2 was used allowing two mismatches in a 25 bp seed and excluding reads with more than 200 alignments (Langmead et al., 2009). RSEM v 1.3.0 was used to estimate isoform or gene relative expression levels in units of "transcripts per million" (tpm), as well as posterior mean estimate of the "expected counts" (the non-normalized absolute number of reads assigned by RSEM to each isoform/gene) (Li and Dewey, 2011; Li et al., 2010). R statistical environment (R core team, 2014) was used at all stages of downstream data analysis.

Downstream RNAseq bioinformatics analysis

Testing for differential expression

The entire set of libraries was pre-normalized as a pool using median normalization routine from EBSeq package (Leng et al., 2013). For each gene, maximal counts across all samples were plotted and the genes representing the lower mode of the distribution were filtered out (only genes that have at least 40 – counts in at least 1 sample were retained, restricting the set of genomic features to 12,635. Additional median scaling was applied to the pre-filtered set of genes. Differential expression was called using EBSeq with 10 iterations. The EBSeq's default procedure of filtering low-expressed genes was suppressed by setting the QtrmCut parameter to zero. Genes with assigned value of Posterior Probability of Differential Expression above 0.95 were preliminary selected. Genes that additionally passed fold change cutoff of 1.5 were selected for network analysis.

Construction of GATA2 network

Using the known transcription-target relationships obtained by combining largely complementary data from HTRIdb (Bovolenta et al., 2012) and CellNet (Cahan et al., 2014), we generated combined sets of targets for 950 transcriptional regulators that involve 130,855 individual transcription factor (TF)-target interactions, for regulon analysis. To visualize the cascades of transcriptional regulation that involve influence of active TFs on TF-encoding genes, we restricted the overall regulatory network to TF-target relationships that involve TF-encoding target genes. The resulting "transcriptional backbone" network has 837 regulators and reduced by over an order of magnitude (12,372) individual TF-target relationships. To visualize the core of the relevant regulatory network, we first selected the regulons that a) responded with FDR below 0.01 in any direction in at least 2 out of 5 comparisons described in the text and b) are listed as both regulators and targets in the "transcriptional backbone" network. The resulting network contains 110 nodes and 983 edges. Each node represents both TF and the gene encoding that TF. The double nature of nodes is used to visualize two types of responses: change in regulon activity (estimated with the complete network of 130,855 TF-target relationships) as node color and change in mRNA of the TF-encoding gene as node size.

Gene-sharing network

To generate gene-sharing networks, we collected respective category-gene relationships (e.g. Gene Ontology Cellular Component) and restricted them to genes that were called differentially expressed in at least one of 5 comparisons (see the Results). Then, for each pair of the categories, the number of shared genes was recorded and used to map to the edge width of the resulting network.

Supplemental References

Bovolenta, L.A., Acencio, M.L., and Lemke, N. (2012). HTRIdb: an open-access database for experimentally verified human transcriptional regulation interactions. BMC Genomics *13*, 405.

Cahan, P., Li, H., Morris, S.A., Lummertz da Rocha, E., Daley, G.Q., and Collins, J.J. (2014). CellNet: network biology applied to stem cell engineering. Cell *158*, 903-915.
Grass, J.A., Jing, H., Kim, S.I., Martowicz, M.L., Pal, S., Blobel, G.A., and Bresnick, E.H. (2006). Distinct functions of dispersed GATA factor complexes at an endogenous gene locus. Mol Cell Biol *26*, 7056-7067.
Hou, Z., Jiang, P., Swanson, S.A., Elwell, A.L., Nguyen, B.K., Bolin, J.M., Stewart, R., and Thomson, J.A. (2015). A cost-effective RNA sequencing protocol for large-scale gene expression studies. Sci Rep *5*, 9570.
Kaimakis, P., de Pater, E., Eich, C., Solaimani Kartalaei, P., Kauts, M.L., Vink, C.S., van der Linden, R., Jaegle, M., Yokomizo, T., Meijer, D*., et al.* (2016). Functional and molecular characterization of mouse Gata2-independent hematopoietic progenitors. Blood *127*, 1426-1437.
Langmead, B., Trapnell, C., Pop, M., and Salzberg, S.L. (2009). Ultrafast and memory-efficient alignment of short DNA sequences to the human genome. Genome Biol *10*, R25.
Leng, N., Dawson, J.A., Thomson, J.A., Ruotti, V., Rissman, A.I., Smits, B.M., Haag, J.D., Gould, M.N., Stewart, R.M., and Kendziorski, C. (2013). EBSeq: an empirical Bayes hierarchical model for inference in RNA-seq experiments. Bioinformatics *29*, 1035-1043.
Li, B., and Dewey, C.N. (2011). RSEM: accurate transcript quantification from RNA-Seq data with or without a reference genome. BMC Bioinformatics *12*, 323.
Li, B., Ruotti, V., Stewart, R.M., Thomson, J.A., and Dewey, C.N. (2010). RNA-Seq gene expression estimation with read mapping uncertainty. Bioinformatics *26*, 493-500.
Mali, P., Yang, L., Esvelt, K.M., Aach, J., Guell, M., DiCarlo, J.E., Norville, J.E., and Church, G.M. (2013). RNA-guided human genome engineering via Cas9. Science *339*, 823-826.
Vodyanik, M.A., Yu, J., Zhang, X., Tian, S., Stewart, R., Thomson, J.A., and Slukvin, II (2010). A mesoderm-derived precursor for mesenchymal stem and endothelial cells. Cell Stem Cell *7*, 718-729.

A

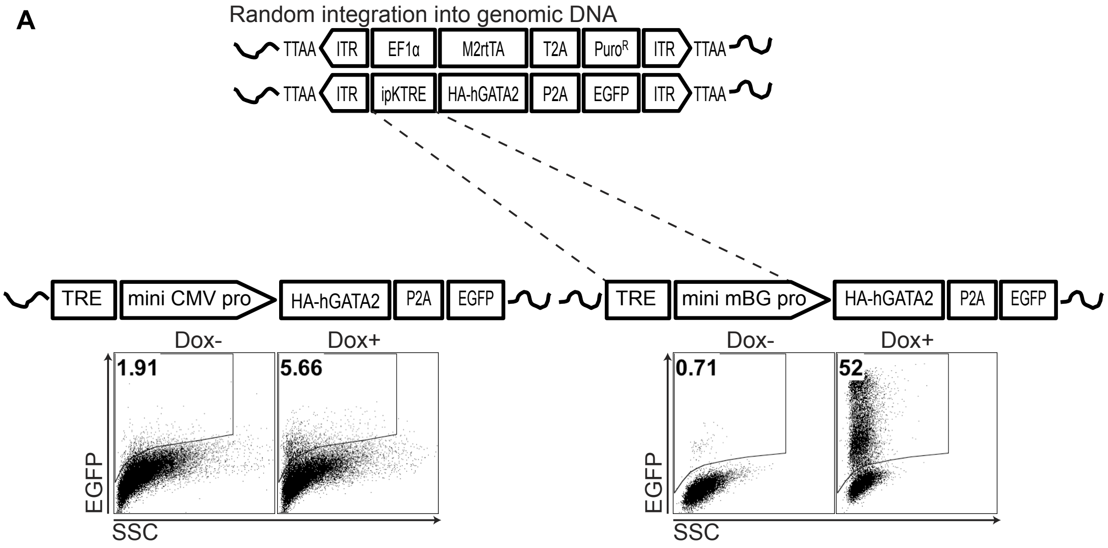

B

Product size 483 bp

```
ATTCCATGGTTCTGAGATGCTTTGATTCCCATGTGAGATTTAGCCCTCCTTGACTGAGCt
||||||||||||||||||||||||||||||||||||||||||||||||||||||||||||
ATTCCATGGTTCTGAGATGCTTTGATTCCCATGTGAGATTTAGCCCTCCTTGACTGAGCT
ggtggggactgggggtggagcgagggtcagggagggggtcgaggtgggcgtgggagTCC
||||||||||||||||||||||||||||||||||||||||||||||||||||||||||||
GGTGGGGACTGGGGGTGGAGCGAGGGTCAGGGAGGGGGTCGAGGTGGGCGTGGGAGTCC
AGCCTGCTGACGCTGCCTTGCCCTCCCAGTCGGCCGCCAGAAGAGCCGGCACCTGTTGTG
||||||||||||||||||||||||||||||||||||||||||||||||||||||||||||
AGCCTGCTGACGCTGCCTTGCCCTCCCAGTCGGCCGCCAGAAGAGCCGGCACCTGTTGTG
CAAATTGTCAGACGACAACCACCACCTTATGGCGCCGAAACGCCAACGGGGACCCTGTCT
||||||||||||||||||||||||||||||||||||||||||||||||||||||||||||
CAAATTGTCAGACGACAACCACCACCTTATGGCGCCGAAACGCCAACGGGGACCCTGTCT
GCAACGCCTGTGGCCTCTACTACAAGCTGCACAATGTGAGTGCGCCCCGCCCCGGCCACC
||||||||||||||||||||||||||||||||||||||||||||||||||||||||||||
GCAACGCCTGTGGCCTCTACTACAAGCTGCACAATGTGAGTGCGCCCCGCCCCGGCCACC
CCGCCCCTCCCAGGGGACCTCTGCGCTTTGTGCTGCCAGGCAAGAGGCCCCAGCCACAAT
||||||||||||||||||||||||||||||||||||||||||||||||||||||||||||
CCGCCCCTCCCAGGGGACCTCTGCGCTTTGTGCTGCCAGGCAAGAGGCCCCAGCCACAAT
ATCCAGCTTGGCTTGGCTTGGGAAGCTGCTGCCCTGAGTGAGCGCCAGAAGGGCTTCCCG
||||||||||||||||||||||||||||||||||||||||||||||||||||||||||||
ATCCAGCTTGGCTTGGCTTGGGAAGCTGCTGCCCTGAGTGAGCGCCAGAAGGGCTTCCCG
TAAGAGGGGTGCCTTGCCTCTGCTCAGGAGGTGGAGCTGGCTAGGACAGGGTCTCGGACT
||||||||||||||||||||||||||||||||||||||||||||||||||||||||||||
TAAGAGGGGTGCCTTGCCTCTGCTCAGGAGGTGGAGCTGGCTAGGACAGGGTCTCGGACT
AGGGAA Genomic DNA for intron-exon5-intron
||||||
AGGGAA PCR products
```

Figure S1. Characterization of GATA2-inducible and GATA2 knockout cell lines, related to Figure 1. (A) Modification of tetracycline response element (ipKTRE), to enhance resistance to transgene silencing during differentiation. Flow cytometry dot plots after 4 days of hematopoietic differentiation show the lack of EGFP expression following DOX treatment of hESCs modified using TREminiCMV promoter. (B) Sequencing of intron-exon5-intron *GATA2* region targeted with gRNA3 and gRNA4 shows preserved structure of this region in generated iG2$^{-/-}$ cells.

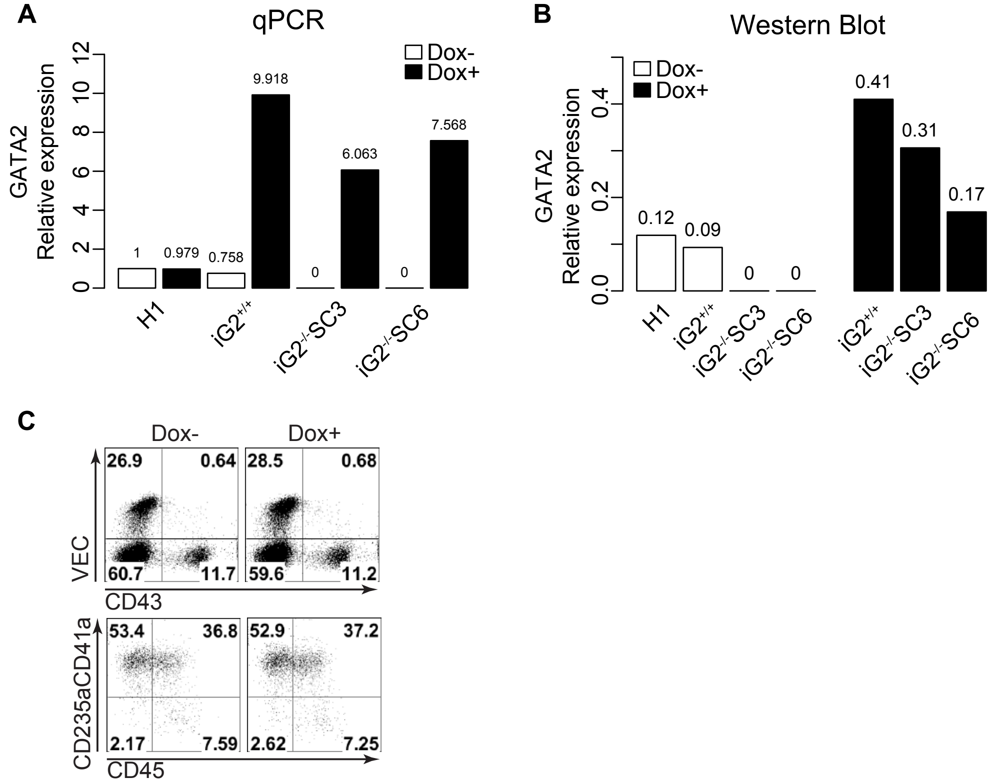

Figure S2. Analysis of DOX effect on GATA2 expression and hematopoietic differentiation, related to Figure 1. (A) qPCR analysis of GATA2 expression on day 5 of hematopoietic differentiation in cultures with or without DOX. (B) Western blot quantification of GATA2 expression on day 5 of hematopoietic differentiation in cultures treated and non-treated with DOX. H1 is wild type H1 hESCs. (C) Flow cytometric analysis blood formation on day 8 of differentiation of wild type H1 hESCs with or without DOX.

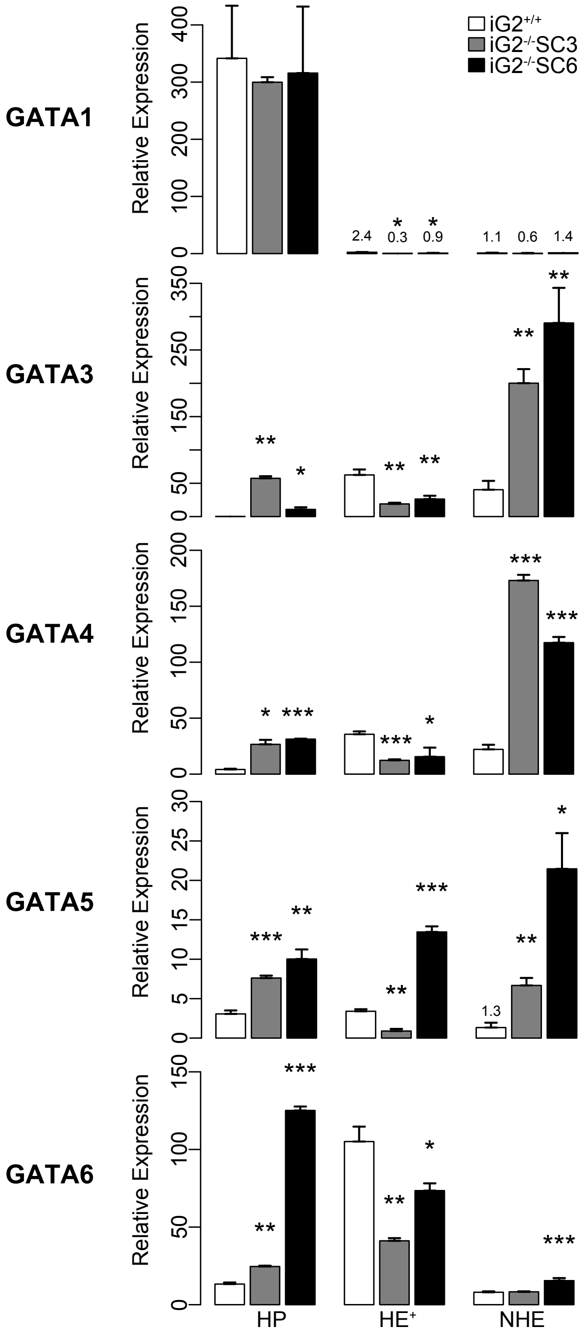

Figure S3. qPCR analysis of GATA factors in CD43^{+} hematopoietic progenitors on day 8 differentiation (HP), HE and non-HE, related to Figure 3. Bars show mean±SE for 3 independent experiments.

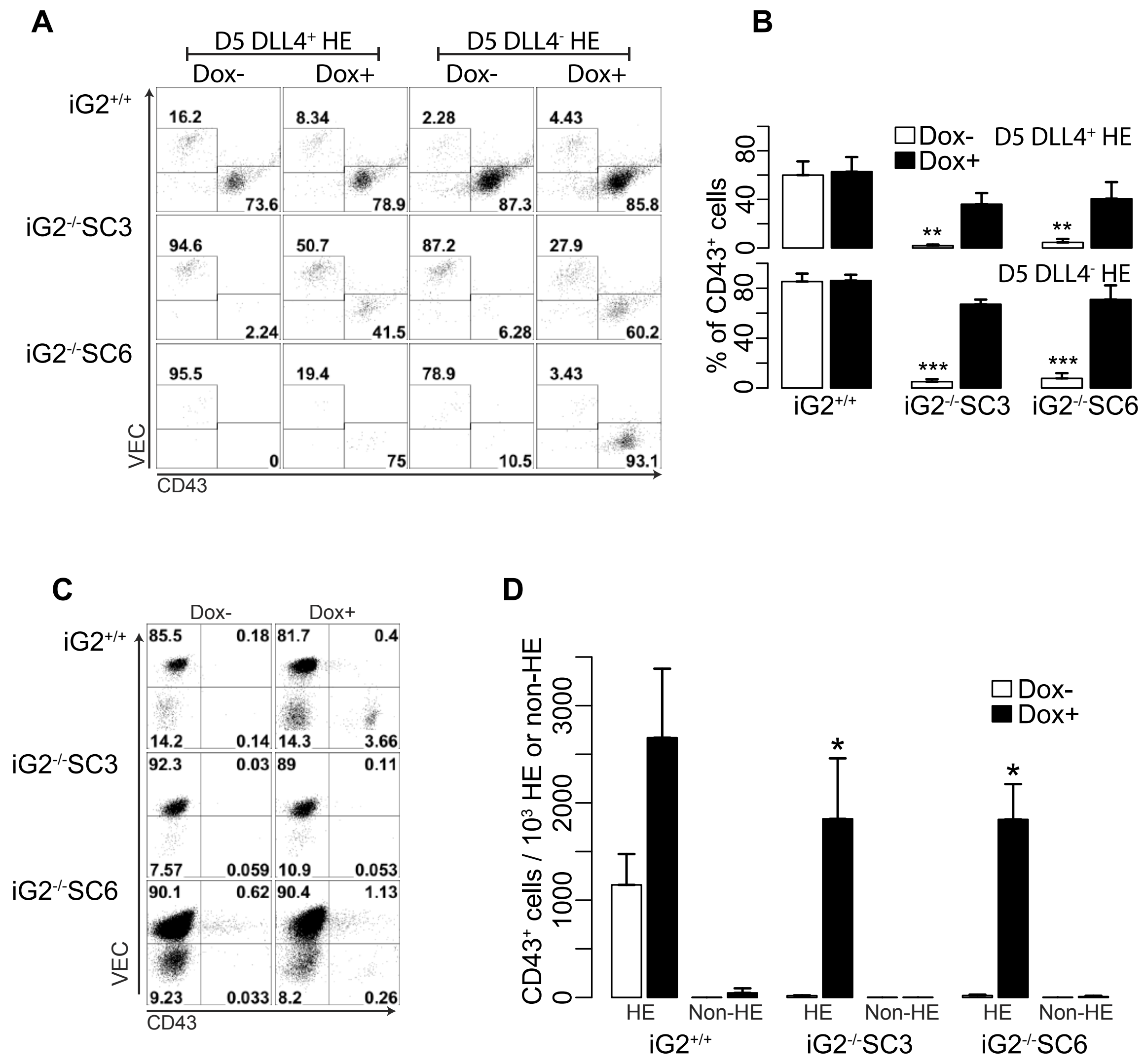

Figure S4. GATA2 effect on blood production from DLL4⁺ and DLL4⁻ HE and non-HE, related to Figure 5. (A) and (B) Blood forming potential of DLL4$^+$ and DLL4$^-$ HE. DLL4$^+$ and DLL4$^-$ VEC$^+$CD43$^-$CD73$^-$ HE cells were isolated by FACS on day 5 of differentiation and cultured on DLL4-OP9 stromal cells with and without DOX for 6 days. (C) Flow cytometric analysis shows the effect of DOX on blood production form VEC$^+$CD43$^-$CD73$^+$ non-HE. Non-HE were isolated from iG2$^{+/+}$ and iG2$^{-/-}$ cells and cultured in HE conditions with or without DOX added during first 2 days of secondary culture. Flow cytometric analysis was performed after 6 days of differentiation. (D) Absolute numbers of CD43$^+$ cells generated from 1000 HE and non-HE cells in DOX+ and DOX- cultures. Bars in (B) and (D) show mean$\pm$SE for 3 independent experiments.

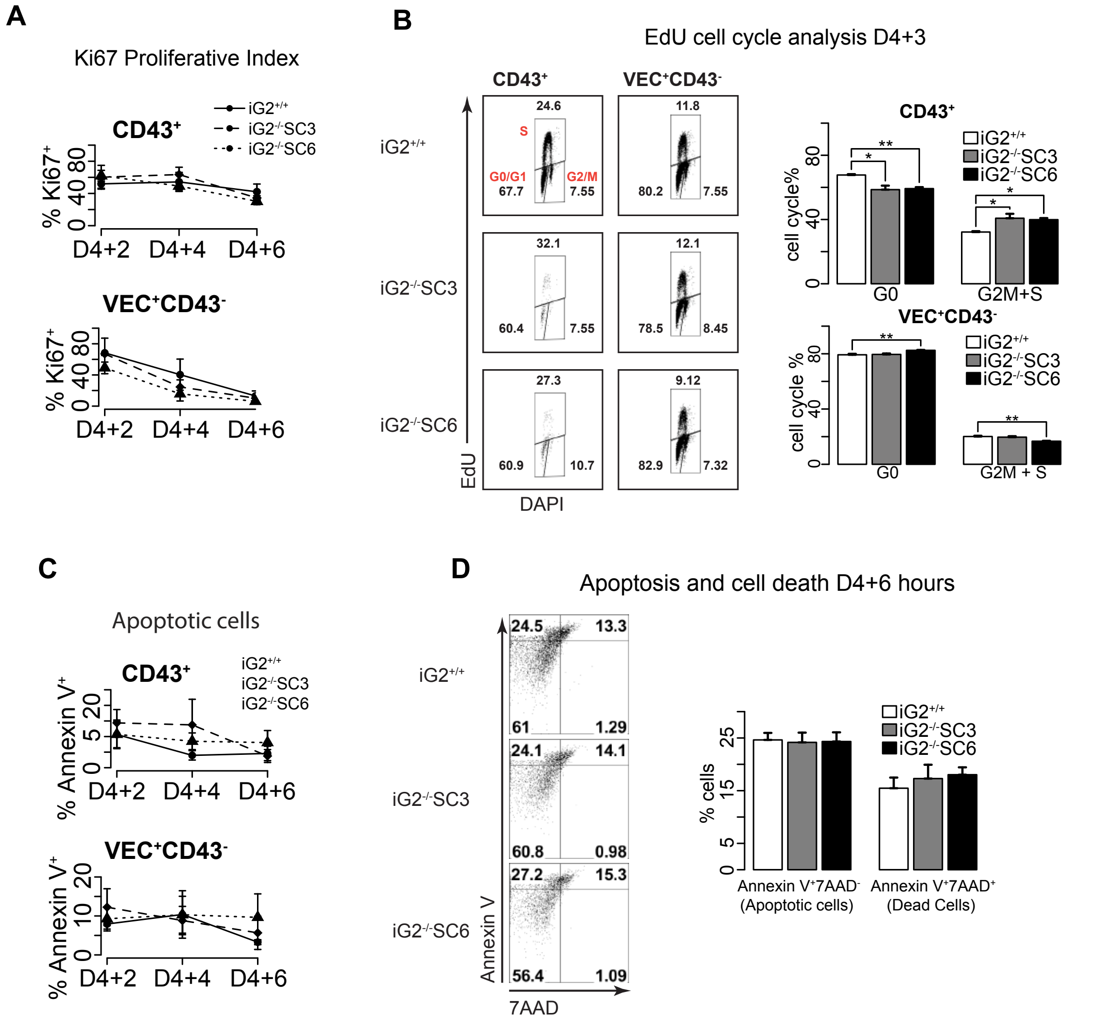

Figure S5. Analysis of proliferative potential and apoptosis in secondary cultures of D4 HE, related to Figure 5. (A) Analysis of cell proliferation in secondary cultures of day 4 (D4) HE using Ki67 immunostaining. Results are mean±SE for 3 independent experiments. (B) Representative dot plots and bar graphs show flow cytometric analysis of cell cycle using EdU and DAPI staining on day 4+3 HE culture. Bar graphs are mean±SE (n=3). (C) Assessment of apoptosis in secondary cultures of day 4 HE using annexin V flow cytometric analysis. (D) Representative dot plots and bar graphs show flow cytometric analysis of apoptosis and necrosis 6 hours post-plating of day 4 HE in secondary cultures. Bar graphs are mean±SE.

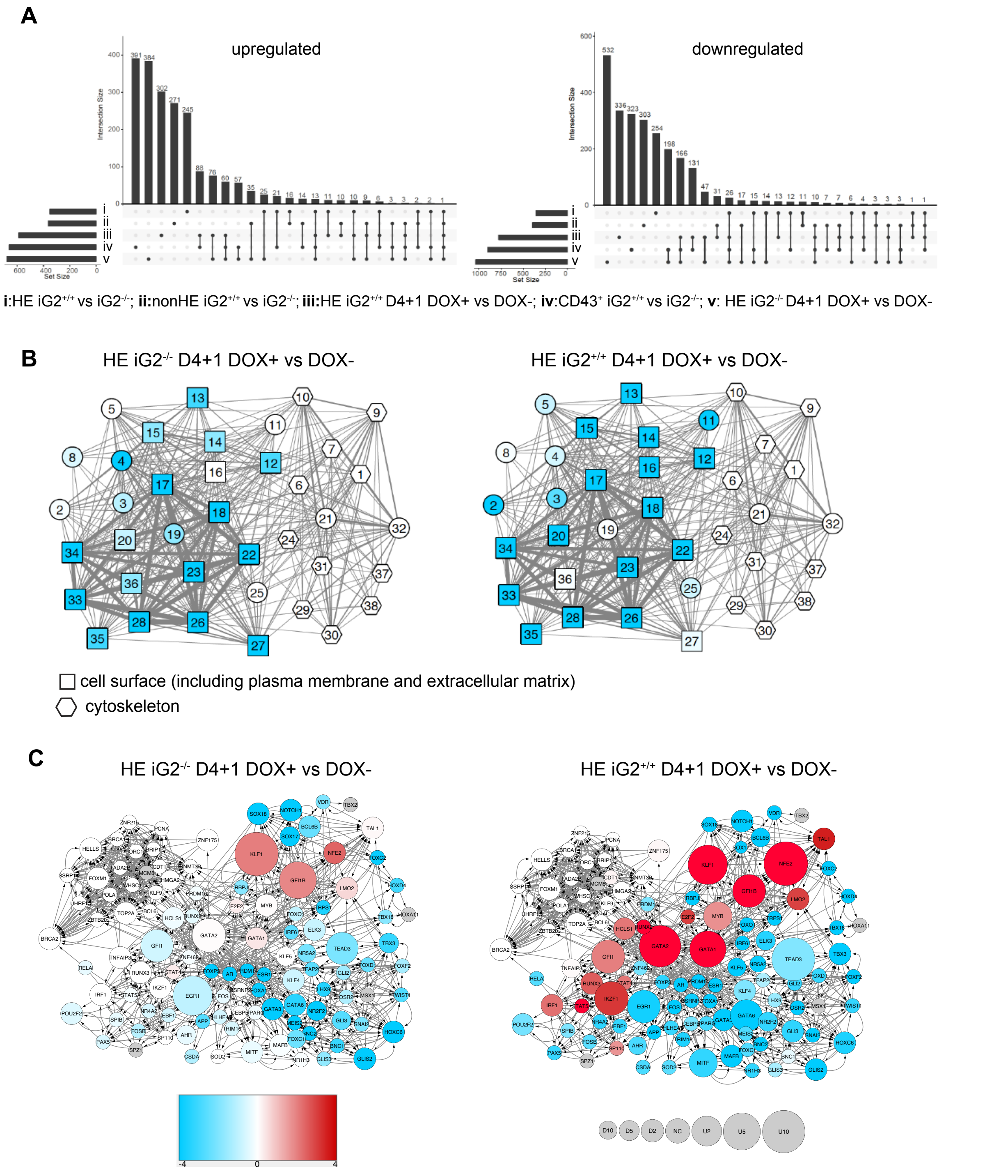

Figure S6. Gene expression profiling reveals distinct features of GATA2 regulatory network during hematopoietic development, related to Figure 6. (A) Bar chart illustrating multi-set intersections of genes upregulated (left chart) and downregulated (right chart) expressed in indicated cell subsets. The combination

matrix marked with circles identifies the genes uniquely upregulated by cell subset/subsets (intersections), while the overlying bars encode the set size and bars on the right show total number of genes uniquely upregulated. (B) GO analysis shows main GOCC categories (cell surface (including plasma membrane and extracellular matrix) and cytoskeleton) found to be affected by GATA2 in indicated cell subsets. Nodes representing those two supercategories are coded by shape, with squares representing cell surface and hexagons representing cytoskeleton. The color density represents enrichment (red) or depletion (blue) of differentially expressed genes related to displayed category. The width of the edges reflects the number of genes shared by categories. The "intracellular non membrane bound organelle" category (#21) that is clearly clustered with the cytoskeleton cohort, is not shape-coded since it does not formally fit the proposed supercategory definition (it encompasses chromosome, cytoskeleton and ribosomes). In our data, ribosome components are not responsive, however chromosome and cytoskeleton-related genes drive enrichment of this category to high significance levels. The node identification numbers correspond to the following GOCC categories: 1 - actin cytoskeleton, 2 - basal lamina, 3 - basement membrane, 4 - cell fraction, 5 - cell projection, 6 – centrosome, 7 - chromosomepericentric region, 8 – collagen, 9 - cytoskeletal part, 10 – cytoskeleton, 11 - dystrophin associated glycoprotein complex, 12 - extracellular matrix, 13 - extracellular matrix part, 14 - extracellular region, 15 - extracellular region part, 16 - extracellular space, 17 - integral to membrane, 18 - integral to plasma membrane, 19 - integrin complex, 20 - intercellular junction, 21 - intracellular non membrane bound organelle, 22 - intrinsic to membrane, 23 - intrinsic to plasma membrane, 24 - kinesin complex, 25 - leading edge, 26 – membrane, 27 - membrane fraction, 28 - membrane part, 29 - microtubule associated complex, 30 - microtubule cytoskeleton, 31 - microtubule organizing center, 32 - non membrane bound organelle, 33 - plasma membrane, 34 - plasma membrane part, 35 - proteinaceous extracellular matrix, 36 - receptor complex, 37 – spindle, 38 - spindle pole. (C) GATA2 transcriptional regulatory network reconstructed based on analysis of differentially expressed genes in iG2$^{+/+}$ and iG2$^{-/-}$ cells. Size of the nodes represents relative abundance of mRNA of the respective gene, computed as $\log_2$(fold change) in iG2$^{+/+}$ versus iG2$^{-/-}$ cells. Both up- and downregulation effects are mapped onto the node size. The color density represents enrichment (red) or depletion (blue) of known targets of that transcription factor (regulon members) among the differentially expressed genes. Network visualization was performed using Cytoscape ver. 3.4.0.

Table S1. Expression of typical HE and non-HE-enriched genes in iG2$^{+/+}$ and iG2$^{-/-}$ HE and non-HE cells and CD43^{+} HPs, Related to Figure 4.

Genes	iG2$^{+/+}$HE	iG2$^{-/-}$HE	iG2$^{+/+}$Non-HE	iG2$^{-/-}$Non-HE	iG2$^{+/+}$CD43	iG2$^{-/-}$CD43
RHAG	23.49	28.22	0.93	1.01	256.16	13.38
GFI1	89.36	65.5	2.64	1.16	10.83	6.38
RUNX1	24.26	11	7.31	3.56	57.57	62.17
NTS	377.13	149.51	32.95	21.22	95.89	17.25
BMPER	140.44	66.38	29.63	34.7	21.5	30.33
SOX17	5.36	21.15	76.98	59.46	0.19	22.59
COL15A1	3.24	6.37	96.78	94.76	2.11	158.94
CAV1	2.38	6.55	16.12	17.21	0.92	7.46
SCG5	0.26	0	14.98	21.27	1.86	1.74
NT5E	0.08	0.24	71.82	35.36	1.25	24.69
EMCN	0.49	1.24	42.37	32.88	0.45	12.76

Table S2. Genomic DNA-PCR for gRNA-mediated mutation screening Related to Experimental Procedures.

**Primers**	**Sequence (5' → 3')**	**Amplified region**	**Product Size (bp*)**
Forward	TGCTCTTTCTCGCCGGATCT	Exon 2	515
Reverse	CCCGCCCCAATTTTTCAGCA		
Forward	CCATGGTTCTGAGATGCTTTG	Exon 5	
Reverse	TCTTACGGGAAGCCCTTCTG		422
Reverse	TTCCCTAGTCCGAGACCCTG		483

*base pairs

Table S3. Primers for qRT-PCR, Related to Experimental Procedure

Genes	Primers	Sequence (5' → 3')	Product Size (bp)
GATA1	Forward	ACCTCCTGACCCTGGGACCT	307
	Reverse	TTCTTGGGCCGGATGAGGGG	
GATA2	Forward	GCGTCTCCTACAGCCCCGCGCAC	360
	Reverse	GGTGCTAGGGTCAGGAGACACTTC	
GATA3	Forward	CTGCCGGAGGAGGTGGATGTGC	248
	Reverse	GGACGTCTTGGAGAAGGGGCTGAG	
GATA4	Forward	GTGCCAACTGCCAGACCACCACC	341
	Reverse	CTGGGACACGGAGCTGCTGTGCC	
GATA5	Forward	TACCACAAGATGAATGGCGT	221
	Reverse	TTCCGTGTCTGGATGCTTTC	
GATA6	Forward	GAGGGAATTCAAACCAGGA	168
	Reverse	CCCTGAGGCTGTAGGTTGTGTTG	
RPL13a	Forward	CCACCCTGGAGGAGAAGAGG	138
	Reverse	CGTGGGTCTTGAGGACCTCTG	
GAPDH	Forward	TTCCAATATGATTCCACCCA	106
	Reverse	GATCTCGCTCCTGGAAGATG	
